# Association between mirtazapine use and serious self-harm in people with depression: an active comparator cohort study using UK electronic health records

**DOI:** 10.1136/ebmental-2021-300355

**Published:** 2022-03-04

**Authors:** Rebecca M Joseph, Ruth H Jack, Richard Morriss, Roger David Knaggs, Debbie Butler, Chris Hollis, Julia Hippisley-Cox, Carol Coupland

**Affiliations:** 1 Centre for Academic Primary Care, Lifespan and Population Health, University of Nottingham School of Medicine, Nottingham, UK; 2 Mental Health & Cognitive Neuroscience, University of Nottingham, Nottingham, UK; 3 NIHR Nottingham Biomedical Research Centre, Nottingham University Hospitals NHS Trust, Nottingham, Nottingham, UK; 4 NIHR MindTech MedTech Co-operative, The Institute of Mental Health, University of Nottingham, Nottingham, UK; 5 School of Pharmacy, University of Nottingham, Nottingham, UK; 6 Nuffield Department of Primary Care Health Sciences, University of Oxford, Oxford, UK

**Keywords:** suicide & self-harm, depression & mood disorders, adult psychiatry

## Abstract

**Background:**

Studies report an increased risk of self-harm or suicide in people prescribed mirtazapine compared with other antidepressants.

**Objectives:**

To compare the risk of serious self-harm in people prescribed mirtazapine versus other antidepressants as second-line treatments.

**Design and setting:**

Cohort study using anonymised English primary care electronic health records, hospital admission data and mortality data with study window 1 January 2005 to 30 November 2018.

**Participants:**

24 516 people diagnosed with depression, aged 18–99 years, initially prescribed a selective serotonin reuptake inhibitor (SSRI) and then prescribed mirtazapine, a different SSRI, amitriptyline or venlafaxine.

**Main outcome measures:**

Hospitalisation or death due to deliberate self-harm. Age–sex standardised rates were calculated and survival analyses were performed using inverse probability of treatment weighting to account for baseline covariates.

**Results:**

Standardised rates of serious self-harm ranged from 3.8/1000 person-years (amitriptyline) to 14.1/1000 person-years (mirtazapine). After weighting, the risk of serious self-harm did not differ significantly between the mirtazapine group and the SSRI or venlafaxine groups (HRs (95% CI) 1.18 (0.84 to 1.65) and 0.85 (0.51 to 1.41) respectively). The risk was significantly higher in the mirtazapine than the amitriptyline group (3.04 (1.36 to 6.79)) but was attenuated after adjusting for dose.

**Conclusions:**

There was no evidence for a difference in risk between mirtazapine and SSRIs or venlafaxine after accounting for baseline characteristics. The higher risk in the mirtazapine versus the amitriptyline group might reflect residual confounding if amitriptyline is avoided in people considered at risk of self-harm.

**Clinical implications:**

Addressing baseline risk factors and careful monitoring might improve outcomes for people at risk of serious self-harm.

**Summary box**

**What is already known about this subject?**

- Some studies have reported an increased risk of self-harm or suicide among people prescribed mirtazapine compared to people prescribed other antidepressants.

**What are the new findings?**

- In this UK-based cohort study of adults with depression prescribed a second-line antidepressant, the rate of serious self-harm (self-harm leading to hospitalisation or death) was higher in people prescribed mirtazapine or venlafaxine compared to people prescribed a selective serotonin reuptake inhibitor (SSRI) or amitriptyline.
- Baseline characteristics differed between the groups, for example people prescribed mirtazapine were more likely to be male and had higher rates of current smoking and heavy drinking compared to the other groups.
- When baseline characteristics were accounted for, the risk of serious self-harm was similar between people prescribed mirtazapine and people prescribed an SSRI or venlafaxine.

**How might it impact clinical practice in the forseeable future?**

- When prescribing antidepressants, discussion of and additional support for risk factors for serious self-harm may improve outcomes for people at risk.

## Introduction

Mirtazapine is licensed in the UK to treat depression in adults.^1^ Although mirtazapine has similar efficacy and tolerability to other antidepressants,^2^ observational studies have suggested an increased risk of self-harm or suicide among people prescribed mirtazapine compared with other antidepressants.^3–6^ Wu *et al* reported a 47% increased risk of hospitalisation for self-harm among people prescribed mirtazapine compared with selective serotonin reuptake inhibitors (SSRIs).^3^ Coupland *et al* reported an increased risk of self-harm for adults prescribed mirtazapine compared with citalopram,^4 5^ and an increased risk of suicide for those aged 20–64 years prescribed mirtazapine compared with citalopram.^4^

Deliberate self-harm and suicidal behaviour are complex health problems associated with a range of risk factors. Prior self-harm is a strong predictor of future self-harm or suicide,^7^ and people with other physical and mental health conditions also have an increased risk.^8^ Some characteristics associated with self-harm or suicide may also influence the choice of antidepressant prescribed, confounding the relationship between individual antidepressants (eg, mirtazapine) and self-harm. Treating underlying mental health conditions, including alcohol and drug use disorders, depression, psychosis and schizophrenia, borderline personality disorder and bipolar disorder, is one of the recommendations of the UK’s National Institute for Health and Care Excellence (NICE) for the long-term management of self-harm,^9^ and antidepressant treatment has been associated with reduced suicide risk in adults with depression.^10^

In the UK, mirtazapine is not recommended as a first-line treatment for depression. NICE recommend that if adults with depression initiate antidepressant treatment, they should be prescribed an SSRI in the first instance, unless contraindicated.^11 12^ The guidelines recommend switching to a different SSRI or an antidepressant from a different class if symptoms have not adequately responded to initial pharmacological interventions. In practice, therefore, people prescribed mirtazapine will often have switched from a different antidepressant.

### Study aim

This study aimed to compare the risk of serious self-harm (suicide or hospital admission due to deliberate self-harm) in people with depression prescribed mirtazapine, an SSRI, amitriptyline or venlafaxine as a second-line antidepressant, accounting for differences in baseline characteristics.

## Methods

The study protocol,^13^ code lists (https://clinicalcodes.rss.mhs.man.ac.uk) and statistical code used to prepare and analyse the data (https://doi.org/10.5281/zenodo.4779024) are available online. The study used anonymised data provided under licence by the Clinical Practice Research Datalink (CPRD). The protocol was reviewed and approved by CPRD’s Independent Scientific Advisory Committee (reference 19_241).

### Data sources

CPRD contains anonymised UK primary care electronic health records, linked to the English Hospital Episode Statistics (HES) data sets and the Office for National Statistics (ONS) mortality data set. The primary care data include coded information about diagnoses, lifestyle characteristics, prescriptions, test results and referrals to secondary care. The November 2019 release of the CPRD GOLD data set was used.

This study used linked hospital admissions and mortality data (set 17, containing records up to 30 November 2018). Linkage to HES data sets and the ONS mortality data set is performed for CPRD by a trusted third party based on National Health Service number, sex, date of birth and postcode. The HES admission data include all diagnoses recorded during an inpatient stay in hospital. The ONS mortality data set includes the date and underlying cause of death.

### Study cohort

People in the cohort had at least 1 year of ‘up-to-standard’ (a data quality indicator) follow-up within CPRD before their first antidepressant prescription and were registered at general practices in England linked to the HES and ONS data sets. The study window was 1 January 2005 to 30 November 2018. People were included if their first recorded antidepressant was an SSRI and was prescribed during the study window, and if they were subsequently prescribed mirtazapine, a different SSRI, amitriptyline or venlafaxine as a second antidepressant at least 1 day after the initial SSRI prescription. The initial prescription date for this second antidepressant was the index date. The index date had to be during or less than 90 days after a period of exposure to the first antidepressant (see ‘Exposure’, below). The people included were aged 18–99 years at their index date and had a record of depression on or before the index date, but no more than 12 months before the first recorded antidepressant prescription. Diagnostic codes (Read v2 codes) for depression were based on existing published code lists (see the online supplemental file). People were excluded if they had a record of bipolar disorder or schizophrenia on or before their index date or had a hospital record of serious self-harm on or before their index date.

10.1136/ebmental-2021-300355.supp1 Supplementary data

People were followed-up from the date of starting their second antidepressant until the earliest of: their first serious self-harm event, stopping the antidepressant (see below), death, leaving their general practice, last data collection date, 30 November 2018, or being prescribed a third antidepressant.

### Exposure

Primary care prescription records were used to estimate exposure to the antidepressants of interest. A published algorithm^14^ which uses information such as daily dose and quantity prescribed was used to estimate the length of each prescription. Further details are provided in the online supplemental file 1.

A risk carry-over window of 30 days was added to the end of each prescription for the study antidepressants. The exposure period ended after this window if there was no new prescription for the drug of interest within that time.

Antidepressant dose was estimated for each active prescription period based on the strength and daily dose of the drugs. Doses were converted to defined daily dose (DDD) using values from the WHO searchable index.^15^

### Outcome

Serious self-harm was defined as a record of ‘intentional self-harm’ (International Classification of Diseases (ICD)-10 codes X60-X84.9) in the hospital admission data, or intentional self-harm as the underlying cause of death in the mortality data. For hospital records, hospitalisation admission date was used as the event date. The earliest recorded event was used.

### Covariates

Covariates were defined with respect to the index date and included age, sex, practice region, body mass index (BMI), smoking status, alcohol intake, socioeconomic status (SES, quintile of the Townsend score)^16^ and ethnicity. Comorbidities and health indicators that might influence choice of antidepressant were defined, including factors in the Charlson comorbidity index^17^ and the QMortality risk prediction algorithm^18^ and prescriptions for other medicines. Where possible code lists were sourced from the CALIBER phenotype resource,^19^ the ClinicalCodes repository^20^ or other published papers. Mental health indicators included depression severity, prior contact with mental health services and a prior primary care record for self-harm. A full list of covariates and further details about how they were defined are available in the online supplemental file 1.

Comorbidities and health indicators were classified as present/not present if recorded on or before index date. Prescribed medicine was classified as present/not present if there was a prescription on or in the 6 months prior to index date. Index year, current and most recent SSRI dose at index date and the length of time between starting the first and second antidepressants were also defined.

### Analysis

Differences in baseline characteristics between the four antidepressant exposure groups were compared using χ^2^ and Kruskal-Wallis tests. Age–sex standardised incidence rates of serious self-harm were calculated using direct standardisation and the age–sex structure of the whole study population. Rate differences between the mirtazapine and other treatment groups were calculated. Survival analysis using Fine-Gray regression^21^ to account for non-suicide death as a competing risk was performed to compare the risk of serious self-harm between the mirtazapine group and the other treatment groups. The proportional hazards assumption was tested by examining log-log plots of survival and comparing observed and predicted Kaplan Meier survival plots. A separate analysis was performed to assess the impact of current antidepressant dose, including current dose of mirtazapine, SSRIs, amitriptyline and venlafaxine as time-varying variables.

Stabilised inverse probability of treatment weighting was used to account for differences in baseline characteristics between the treatment groups. Propensity scores were estimated using multinomial logistic regression. From these, inverse weights were calculated, then stabilised by multiplying by the unadjusted probability of being in the groups.^22^ The propensity score model was assessed using goodness-of-fit tests, examining overlap in propensities graphically and testing the balance of each covariate after weighting. Information about the final model is provided in online supplemental table S1.

Multiple imputation by chained equations was used to estimate missing values of BMI, ethnicity, smoking status, alcohol intake and SES. The imputation models included all variables used to estimate the propensity scores, the study outcome and follow-up time. Twenty imputed data sets were generated. The regression analyses were performed on each imputed data set and then the results were combined using Rubin’s rules.^23 24^

In line with the protocol, we reported statistical significance at the 0.05 level. Results are presented with 95% CIs. Data analyses were performed using Stata MP/V.16.1.

### Sensitivity analyses

Rates were recalculated after excluding people with baseline primary care records of self-harm, and including people with baseline schizophrenia or bipolar disorder. Regression analyses were repeated: using multivariable Fine-Gray regression, using Cox regression, and using all defined covariates to estimate propensity scores. Further sensitivity analyses using multivariable Cox regression were excluding people with primary care records of self-harm at baseline; stratifying by age group (18–64 years, 65–99 years), changing the risk carry-over window (0 days, 6 months and the end of follow-up); censoring follow-up after 1 year or 5 years and restricting the first or second SSRI to citalopram, the most commonly prescribed SSRI (see online supplemental table S2). Finally, we included primary care records for self-harm in the definition of the outcome, excluding people with a baseline primary care record of self-harm.

### Patient and public involvement

The study team included two patient and public involvement representatives who contributed to discussions at all stages of the study, one of whom (DB) helped author this paper. We also discussed the study with the National Institute for Health Research MindTech Involvement Team, a group of individuals with lived experience of mental health conditions.

## Results

**Figure 1:**
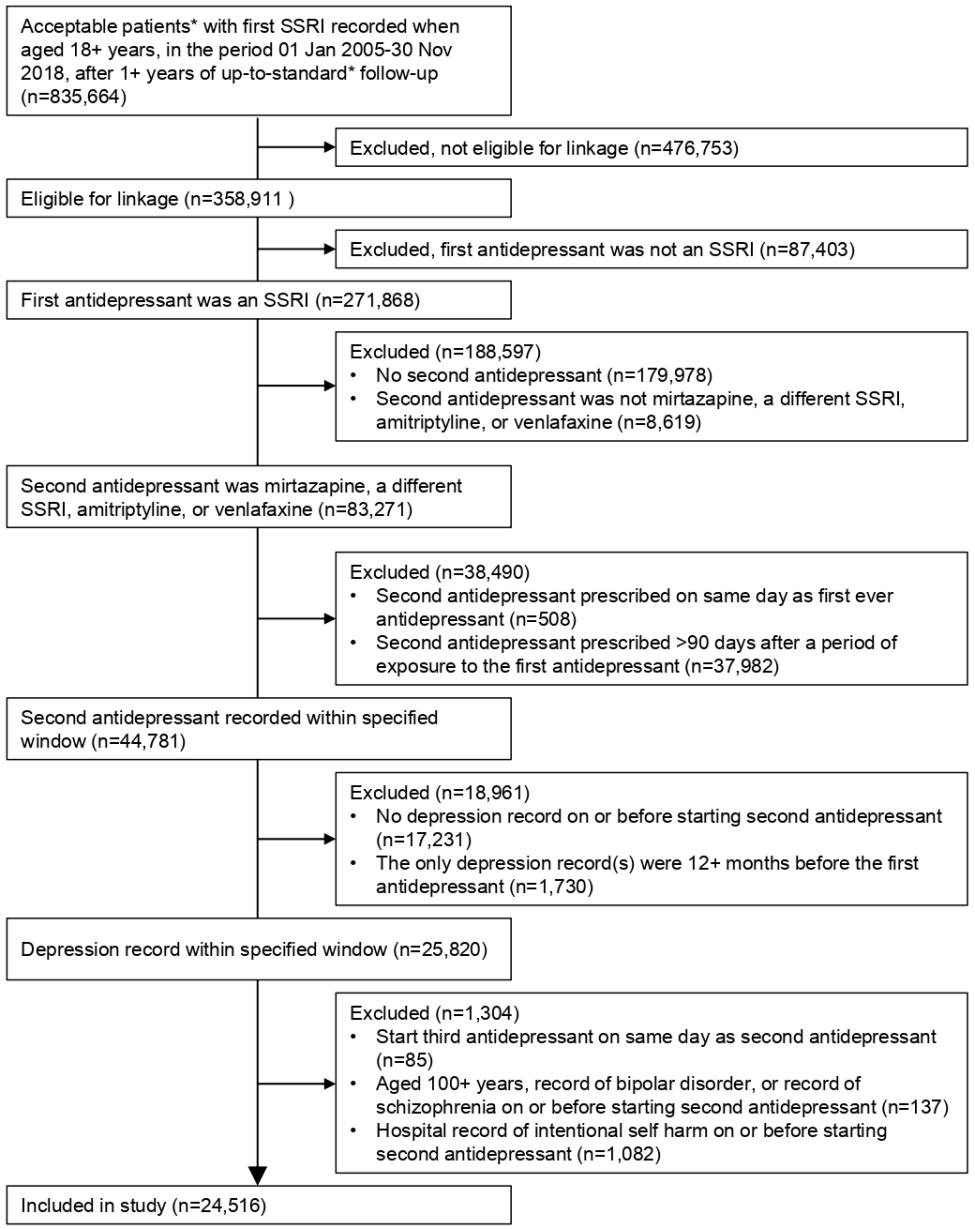
Flow diagram showing the definition of the study cohort. SSRI, selective serotonin reuptake inhibitor. *Markers of data quality in the Clinical Practice Research Datalink.

Figure 1 shows the selection of the study cohort. Of the 358 911 people for whom linked data were requested, 24 516 people from 380 general practices met the inclusion criteria: 4777 (19.5%) people in the mirtazapine group, 14 428 (58.8%) in the SSRI group, 3801 (15.5%) in the amitriptyline group and 1510 (6.2%) in the venlafaxine group.

Baseline characteristics are summarised in table 1, with additional results in online supplemental table S2. The median age of the study population was 41 years (IQR, 29–54 years). A higher proportion of the mirtazapine group was men (51.4%, compared with 41.6% for the whole study population). The mirtazapine group had the lowest median BMI (25.6) and the highest proportion of current smokers (34.9%) and heavy drinkers (6.1%).

**Table 1:** Characteristics of people in the study cohort, determined at index date

	All	Mirtazapine	SSRI	Amitriptyline	Venlafaxine	Statistic
**Count**	24 516	4777	14 428	3801	1510	
**Age, median (IQR**)	41 (29–54)	44 (31–59)	39 (27–51)	48 (37–61)	41 (30–51)	KW χ^2^(3)=890.9, p<0.001
**Sex, n (%**)						
Male	10 190 (41.6)	2456 (51.4)	5731 (39.7)	1303 (34.3)	700 (46.4)	
Female	14 326 (58.4)	2321 (48.6)	8697 (60.3)	2498 (65.7)	810 (53.6)	χ^2^(3)=308.3, p<0.001
Ethnicity, n (%)*						
Asian	447 (2.5)	105 (2.9)	237 (2.3)	83 (2.8)	22 (2.1)	
Black	259 (1.5)	37 (1.0)	150 (1.5)	56 (1.9)	16 (1.5)	
Mixed	159 (0.9)	35 (1.0)	96 (0.9)	18 (0.6)	10 (1.0)	
Other	214 (1.2)	34 (1.0)	136 (1.3)	32 (1.1)	12 (1.1)	
White	16 728 (93.9)	3350 (94.1)	9614 (94.0)	2773 (93.6)	991 (94.3)	χ^2^(12)=21.1, p=0.049
**Missing ethnicity, n (%**)	6709 (27.4)	1216 (25.5)	4195 (29.1)	839 (22.1)	459 (30.4)	χ^2^(3)=90.5, p<0.001
Townsend Score quintile, n (%)*						
1 (least deprived)	4830 (19.7)	861 (18.0)	2853 (19.8)	795 (20.9)	321 (21.3)	
2	4962 (20.3)	941 (19.7)	2898 (20.1)	763 (20.1)	360 (23.9)	
3	5290 (21.6)	1004 (21.0)	3118 (21.6)	843 (22.2)	325 (21.5)	
4	5305 (21.7)	1049 (22.0)	3186 (22.1)	807 (21.2)	263 (17.4)	
5 (most deprived)	4111 (16.8)	918 (19.2)	2360 (16.4)	593 (15.6)	240 (15.9)	χ^2^(12)=60.1, p<0.001
**Missing Townsend score, n (%**)	30 (0.1%)‡	<5	13 (0.1%)	<5	<5	–
BMI, median (IQR)*	26.2 (22.8–30.8)	25.6 (22.4–29.8)	26.1 (22.7–30.5)	27.2 (23.5–32.2)	26.7 (23.2–31.2)	KW χ^2^(3)=136.6, p<0.001
**Missing BMI, n (%**)	6954 (28.4)	1409 (29.5)	4316 (29.9)	794 (20.9)	435 (28.8)	χ^2^(3)=124.7, p<0.001
Smoking status, n (%)*						
Never	9635 (40.5)	1774 (38.3)	5725 (41.0)	1503 (40.1)	633 (43.4)	
Former	6565 (27.6)	1243 (26.8)	3747 (26.8)	1173 (31.3)	402 (27.6)	
Current	7600 (31.9)	1614 (34.9)	4496 (32.2)	1068 (28.5)	422 (29.0)	χ^2^(6)=62.7, p<0.001
**Missing smoking status, n (%**)	716 (2.9)	146 (3.1)	460 (3.2)	57 (1.5)	53 (3.5)	χ^2^(3)=32.9, p<0.001
Alcohol intake, n (%)*						
Non-drinker	3200 (33.4)	615 (31.8)	1875 (34.3)	534 (32.3)	176 (34.1)	
Former drinker	1367 (14.3)	316 (16.3)	725 (13.3)	263 (15.9)	63 (12.2)	
Occasional drinker	4092 (42.7)	790 (40.8)	2345 (42.9)	733 (44.3)	224 (43.4)	
Moderate drinker	450 (4.7)	98 (5.1)	256 (4.7)	71 (4.3)	25 (4.8)	
Heavy drinker	464 (4.8)	118 (6.1)	264 (4.8)	54 (3.3)	28 (5.4)	χ^2^(12)=36.8, p<0.001
**Missing alcohol intake, n (%**)	14 943 (61.0)	2840 (59.5)	8963 (62.1)	2146 (56.5)	994 (65.8)	χ^2^(3)=60.1, p<0.001
**Mental health indicators**						
Severe depression†, n (%)	2303 (9.4)	472 (9.9)	1323 (9.2)	327 (8.6)	181 (12.0)	χ^2^(3)=16.9, p=0.001
Recorded depression scale, n (%)	15 076 (61.5)	2862 (59.9)	8879 (61.5)	2383 (62.7)	952 (63.0)	χ^2^(3)=8.9, p=0.031
Alcohol misuse, n (%)	768 (3.1)	220 (4.6)	407 (2.8)	99 (2.6)	42 (2.8)	χ^2^(3)=42.9, p<0.001
Anxiety, n (%)	7319 (29.9)	1458 (30.5)	4292 (29.7)	1090 (28.7)	479 (31.7)	χ^2^(3)=6.1, p=0.106
Contact with mental health services, n (%)	5895 (24.0)	1488 (31.1)	3264 (22.6)	684 (18.0)	459 (30.4)	χ^2^(3)=257.5, p<0.001
Eating disorder, n (%)	94 (0.4)	17 (0.4)	61 (0.4)	9 (0.2)	7 (0.5)	χ^2^(3)=3.1, p=0.380
Insomnia, n (%)	2981 (12.2)	769 (16.1)	1411 (9.8)	621 (16.3)	180 (11.9)	χ^2^(3)=208.1, p<0.001
Intellectual disability, n (%)	80 (0.3)‡	8 (0.2)	58 (0.4)	9 (0.2)	<5	χ^2^(3)=7.4, p=0.059
Personality disorder, n (%)	101 (0.4)	24 (0.5)	52 (0.4)	15 (0.4)	10 (0.7)	χ^2^(3)=4.2, p=0.239
Self-harm (primary care), n (%)	863 (3.5)	185 (3.9)	507 (3.5)	110 (2.9)	61 (4.0)	χ^2^(3)=7.3, p=0.062
Substance misuse disorder, n (%)	577 (2.4)	177 (3.7)	303 (2.1)	65 (1.7)	32 (2.1)	χ^2^(3)=49.2, p<0.001

*Counts and percentages do not include missing values.

†Severe depression: Either a Read code for severe depression or depression with psychosis, scoring 15 or above on the Patient Health Questionnaire-9 scale, or scoring 16 or above on the Hospital Anxiety and Depression scale.

‡Value rounded to mask small numbers.

BMI, body mass index; KW, Kruskal-Wallis test; n, number; SSRI, selective serotonin reuptake inhibitor.

The median length of follow-up ranged from 2.2 (IQR 1.9–5.2) months (amitriptyline group) to 5.6 (IQR 2.0–21.3) months (venlafaxine group) (online supplemental table S3). Overall, there were 235 serious self-harm events (including 13 deaths) over 26 679 person-years of follow-up, giving a crude incidence rate of 8.8 (95% CI 7.8 to 10.0) events/1000 person-years. Age–sex standardised rates are summarised in table 2. The mirtazapine group had the highest standardised rate of serious self-harm (14.1 events/1000 person-years, 95% CI 10.4 to 18.7), with 6.1 additional events/1000 person-years compared with the SSRI group and 10.3 additional events/1000 person-years compared with the amitriptyline group.

**Table 2:** Crude and age-sex standardised rates of serious self-harm (per 1000 person-years)

	Number of events	Person-years	Crude event rate (95% CI)	Standardised event rate (95% CI)	Rate difference (95% CI)
**Total**					
All	235	26 679	8.8 (7.8 to 10.0)	8.8 (7.7 to 10.0)	–
Mirtazapine	57	4434	12.9 (9.9 to 16.7)	14.1 (10.4 to 18.7)	reference
SSRI	143	17 006	8.4 (7.1 to 9.9)	8.0 (6.8 to 9.5)	−6.1 (–7.9 to –4.3)
Amitriptyline	8	3045	2.6 (1.3 to 5.3)	3.8 (1.6 to 7.5)	−10.3 (–11.9 to –8.7)
Venlafaxine	27	2194	12.3 (8.4 to 17.9)	11.7 (7.6 to 18.0)	−2.4 (–4.3 to –0.5)
Men*					
All	118	10 987	10.7 (9.0 to 12.9)	11.1 (9.2 to 13.3)	–
Mirtazapine	–	–	15.0 (10.9 to 20.7)	15.3 (10.7 to 21.0)	reference
SSRI	–	–	10.1 (7.9 to 12.8)	9.7 (7.5 to 12.4)	−5.6 (–8.6 to –2.7)
Amitriptyline	–	–	3.9 (1.5 to 10.3)	5.4 (1.4 to 13.2)	−9.9 (–12.6 to –7.2)
Venlafaxine	–	–	11.9 (6.6 to 21.5)	11.2 (5.5 to 22.2)	−4.1 (–7.1 to –1.1)
Women*					
All	117	15 692	7.5 (6.2 to 8.9)	7.1 (5.9 to 8.5)	–
Mirtazapine	–	–	10.2 (6.6 to 15.8)	13.4 (8.1 to 20.4)	reference
SSRI	–	–	7.4 (5.9 to 9.2)	6.9 (5.4 to 8.6)	−6.5 (–8.7 to –4.3)
Amitriptyline	–	–	2.0 (0.7 to 5.3)	2.8 (0.7 to 7.1)	−10.6 (–12.6 to –8.6)
Venlafaxine	–	–	12.6 (7.7 to 20.6)	12.1 (6.7 to 21.7)	−1.3 (–3.8 to 1.2)

*Numbers in subgroups suppressed due to small numbers.

SSRI, selective serotonin reuptake inhibitor.

Table 3 shows the results of survival analyses using Fine-Gray regression. The proportional hazards assumption was met, although the majority of events happened early in follow-up (67% in the first 6 months). In the propensity score weighted analysis, the risk of serious self-harm in the mirtazapine group was not significantly different to the SSRI group (subdistribution HR (SHR) 1.18, 95% CI 0.84 to 1.65) or the venlafaxine group (SHR 0.85, 95% CI 0.51 to 1.41). The risk of serious self-harm was significantly higher in the mirtazapine group compared with the amitriptyline group (SHR 3.04, 95% CI 1.36 to 6.79).

**Table 3:** Results of Fine-Gray (competing risks) regression comparing the risk of serious self-harm between study treatment groups

	Unadjusted, SHR (95% CI)	Age-sex adjusted, SHR (95% CI)	Propensity score weighted, SHR (95% CI)
Mirtazapine vs SSRI	1.35 (0.99 to 1.84)	1.51 (1.11 to 2.06)	1.18 (0.84 to 1.65)
Mirtazapine vs amitriptyline	5.06 (2.42 to 10.59)	4.33 (2.05 to 9.11)	3.04 (1.36 to 6.79)
Mirtazapine vs venlafaxine	0.84 (0.53 to 1.32)	0.94 (0.59 to 1.48)	0.85 (0.51 to 1.41)

SHR, subdistribution hazard ratio; SSRI, selective serotonin reuptake inhibitor.

After accounting for current antidepressant dose, the difference in risk between the mirtazapine and amitriptyline groups was attenuated, and no longer statistically significant in the weighted model (SHR 1.70, 95% CI 0.59 to 4.85) (online supplemental table S4). The risk of serious self-harm increased with increasing current dose of mirtazapine (not statistically significant in the multivariable adjusted model), SSRIs and venlafaxine.

### Sensitivity analyses

After excluding 863 people with a primary care record of self-harm at baseline, the rate difference between the mirtazapine and the SSRI and amitriptyline groups was reduced. Including people with baseline schizophrenia or bipolar disorder made no difference to the event rates (online supplemental table S5).

Overall, changes to the regression analyses did not have a large impact on the results (online supplemental tables S6 and S7). For the comparison between mirtazapine and amitriptyline, excluding people with baseline primary care self-harm records attenuated the risk difference (HR 2.26, 95% CI 1.04 to 4.88), as did restricting to those aged 18–64 years (HR 2.84, 95% CI 1.33 to 6.06) and including primary care records when defining the self-harm outcome (HR 1.96, 95% CI 1.15 to 3.35). Lengthening the carry-over period after stopping the antidepressant led to a smaller difference in risk (HR 2.08, 95% CI 1.31 to 3.31 when the carry-over window continued to the end of follow-up). For the comparison between mirtazapine and venlafaxine, excluding people with baseline primary care self-harm records increased the risk difference (HR 0.60, 95% CI 0.36 to 1.01). Overall, there were few events in the 65–99 years age group. Restricting to a specific SSRI (citalopram) led to some changes in the magnitudes of the effects, but the sample size was reduced for these comparisons.

## Discussion

Adults with depression who were prescribed mirtazapine as a second-line antidepressant had higher age–sex standardised rates of serious self-harm than people prescribed amitriptyline or an SSRI. However, when baseline covariates were accounted for the risk of serious self-harm in people prescribed mirtazapine was not statistically significantly different to the risk in people prescribed an SSRI or venlafaxine. The risk of serious self-harm remained significantly higher in the mirtazapine group compared with the amitriptyline group, although the difference was attenuated after current antidepressant dose was accounted for.

The difference in risk between the mirtazapine and SSRI group was smaller than that reported in the previous studies^3–5^ and, like Valenstein *et al*,^25^ was not statistically significant. In the sensitivity analysis excluding people with baseline primary care self-harm records the direction of the risk difference was reversed, possibly indicating a higher baseline risk for the mirtazapine group that was not fully accounted for in the main analysis. This study looked at second-line antidepressants, whereas Wu *et al* 3 grouped people according to their first recorded antidepressant and Coupland *et al* 4 allowed the antidepressant exposure groups to vary over time. These differences may account for the difference in results. The ‘new user’ design used in the current study aims to reduce the level of residual or unmeasured confounding by comparing people at a similar point in their disease and treatment history.^26^ We designed the study around new users of the second-line antidepressants to mirror the UK treatment guidelines.^11^ Coupland *et al* 4 included primary care records when defining their study outcome. We performed a sensitivity analysis in which we included primary care self-harm records in our outcome definition. In this sensitivity analysis, the risk difference between the mirtazapine and SSRI groups was a similar magnitude to the main study and was non-significant (online supplemental table S7).

We found a threefold difference in risk between the mirtazapine and amitriptyline groups, but this was based on only eight events in the amitriptyline group. Several factors suggest that the amitriptyline group represents a different underlying population, for example, the higher rates of pain medicine use and conditions such as abdominal and neuropathic pain, and the low average dose of amitriptyline throughout follow-up (median DDD of 0.2—see online supplemental tables S8 and S9) which suggests prescribing for pain rather than depression. In addition, it has been argued that clinicians may avoid prescribing tricyclic antidepressants to people who are at a higher risk of suicide because they more toxic in overdose.^3 4 27^ These differences may not be fully accounted for in the analysis and likely explain at least some of the risk difference.

There has been little direct comparison between mirtazapine or venlafaxine, although there has been some discussion about a potential increased risk of self-harm or suicide in people prescribed venlafaxine compared with SSRIs.^4 28^ In this study, the rate of serious self-harm was similar between the mirtazapine and venlafaxine groups, and the groups did not differ significantly in adjusted analyses. This result was similar to Valenstein *et al*.^25^ The risk difference increased in some of the sensitivity analyses (eg, after excluding people with baseline primary care self-harm records), and it would be interesting to study this with a larger cohort.

### Strengths and limitations

The study was designed to reduce indication and channelling biases—everyone in the study had a diagnostic code for depression, a recent prescription for an SSRI and were new users of the drugs investigated. This improves the likelihood that the results are valid,^26^ but the additional inclusion and exclusion criteria are at the cost of power and generalisability. We may have excluded some people with depression if depression was not recorded or was recorded more than a year before starting antidepressants, or if only depression symptoms were recorded. Regarding generalisability, people in the study cohort were similar in terms of demographic characteristics to all those for whom we requested linked data, that is, people prescribed an SSRI within the study window (online supplemental table S10). As expected, the study cohort had slightly higher depression severity and a smaller proportion had a primary care record for self-harm at baseline. As the study compared second-line antidepressants, the results may not transfer to people prescribed one of the study drugs as their first antidepressant. Based on the basic data used to define the study population, applying only the follow-up date criteria and not considering other antidepressants not included in the study, approximately 19 000 people had mirtazapine as their first recorded antidepressant compared with approximately 18 000 people with mirtazapine as their second recorded antidepressant. Therefore, it is possible that our study findings based on second-line treatment apply to approximately half of mirtazapine users in the UK.

We accounted for baseline covariates using propensity score methods. However, as this was an observational study, there remains a risk of residual and unmeasured confounding. Some of the measures defined in the study may be incompletely captured in the medical record, other risk factors may not be recorded at all, and any differences in likelihood of seeking medical attention could lead to differential reporting of risk factors.

As prescriptions issued in UK general practices are automatically captured in the electronic record, the prescription data in CPRD are generally considered complete records of primary care prescribing.^29^ However, data about secondary care prescribing are not available in the data sets used for the study. Thus, there may be some underestimation of drug use, particularly in people with more severe illness. In addition, the prescription data do not guarantee that a prescription was filled or taken as prescribed. Differential adherence to the study drugs could introduce bias, particularly given the association between depression and self-harm or suicide.^30^

Serious self-harm is a rare outcome and CIs were wide, so we cannot rule out larger risk differences than those found in this study. The study outcome included only ‘intentional’ self-harm, thus may have excluded some true events that were classified as ‘undetermined intent’. The outcome included only the most severe events—those that led to a hospital admission or that were fatal—and so only represents this particular aspect of self-harm and suicidal behaviour.

This analysis differed from the original protocol^13^ in the following ways. First, a 30-day risk carry-over window was used instead of the planned 6 months (included as a sensitivity analysis). The original window was tailored to a different outcome (mortality), an analysis that will be reported separately. Based on the existing studies and the consideration that antidepressant-related self-harm events are thought to occur most frequently around the time of starting or stopping treatment,^4^ the shorter window was used to reduce the level of exposure misclassification. Second, we did not separate those who switched treatment from those who augmented treatment, due to the difficulty in defining this without using future exposure data. Thus, the study groups include some people who continued their original SSRI alongside the new treatment (summarised in online supplemental table S11).

## Conclusions

People with depression prescribed mirtazapine as a second-line antidepressant had a higher age–sex standardised rate of serious self-harm than people prescribed an SSRI, amitriptyline or venlafaxine. However, after accounting for additional baseline characteristics, people prescribed mirtazapine were not at a significantly increased risk of serious self-harm compared with people prescribed an SSRI or venlafaxine. Although we found an increased risk of self-harm for people prescribed mirtazapine compared with amitriptyline, the number of outcomes was low for this comparison, and other factors (eg, channelling bias) could have influenced this result. The higher rate of serious self-harm in people prescribed mirtazapine may reflect the higher prevalence of other risk factors in this group, for example, alcohol misuse. Thus, when prescribing antidepressants, discussion of and additional support for such risk factors may improve outcomes for people at risk of serious self-harm.

## Data Availability

No data are available. Data used in the study were provided under licence by CPRD (www.cprd.com) and cannot be shared by the authors. All code lists and the statistical code (in the form of Stata do-files) used to prepare and analyse the data are available on Zenodo.org (https://doi.org/10.5281/zenodo.4779024).
