# Supplementary material for: Association between mirtazapine use and serious self-harm in people with depression: an active comparator cohort study using UK electronic health records
Source: Evid Based Ment Health. 2022 Mar 4;25(4):169–76. doi: 10.1136/ebmental-2021-300355 (PMC9685727; doi:10.1136/ebmental-2021-300355)
Supplement: Supplementary data [file ebmental-2021-300355supp001.pdf]

Supplementary File

Contents

Additional methodological details 2
- Antidepressant exposure 2
- Variable list and details 3
- Propensity score model 4

Additional results 5
- Supplementary Table S1. Propensity score model 5
- Supplementary Table S2. Full baseline characteristics 6
- Supplementary Table S3. Median follow-up per study group 10
- Supplementary Table S4. Survival analyses comparing the risk of serious self-harm between study treatment groups while accounting for current antidepressant dose 11
- Supplementary Table S5. Sensitivity analyses – rates after altering study inclusion criteria 12
- Supplementary Table S6. Sensitivity analysis – multivariable competing risks regression 13
- Supplementary Table S7. Sensitivity analyses – varying the survival analysis models (all analyses used Cox regression) 14
- Supplementary Table S8. Summary of antidepressant doses during follow-up (including times of non-use) 16
- Supplementary Table S9. Summary of antidepressant doses during follow-up (excluding times of non-use) 16
- Supplementary Table S10. Comparison of characteristics between those meeting the different inclusion criteria 17
- Supplementary Table S11. Number of active antidepressant prescriptions at follow-up=3months, by study treatment group 19

References 20

Joseph RM, *et al. Evid Based Ment Health* 2022; 25:169–176. doi: 10.1136/ebmental-2021-300355

Additional methodological details

Antidepressant exposure

The start and stop dates of individual antidepressant prescriptions were defined using a published algorithm.[1] The start dates are the prescription dates unless they have been adjusted for any overlapping time. Prescription duration is not entered for most prescriptions and must be estimated from other information. The algorithm used includes 10 decision steps, which are shown below with the decisions used highlighted.

Figure modified from Pye S et al 2018[1], which is available through a Creative Commons CC BY licence.

Step:	A. Data cleaning				
Decision node:	1<br>Clean implausible qty	2<br>Clean missing qty	3<br>Clean implausible ndd	4<br>Clean missing ndd	5<br>Clean all available duration variables
	a. Use implausible value	a. Keep as missing	a. Use implausible value	a. Keep as missing	a. Do nothing
	**b. Set to missing**	b1. Set to individual mean<br>b2. Set to practice mean<br>b3. Set to population mean	**b. Set to missing**	b1. Set to individual mean<br>b2. Set to practice mean<br>b3. Set to population mean	**b(6). Set to missing if > 6 months**
	c1. Set to individual mean<br>c2. Set to practice mean<br>c3. Set to population mean	**c1. Set to individual median**<br>c2. Set to practice median<br>c3. Set to population median	c1. Set to individual mean<br>c2. Set to practice mean<br>c3. Set to population mean	**c1. Set to individual median**<br>c2. Set to practice median<br>c3. Set to population median	b(12). Set to missing if >12 months
	d1. Set to individual median<br>d2. Set to practice median<br>d3. Set to population median	d1. Set to individual mode<br>d2. Set to practice mode<br>d3. Set to population mode	d1. Set to individual median<br>d2. Set to practice median<br>d3. Set to population median	d1. Set to individual mode<br>d2. Set to practice mode<br>d3. Set to population mode	b(24). Set to missing if >24 months
	e1. Set to individual mode<br>e2. Set to practice mode<br>e3. Set to population mode	e1. Use individual's next value<br>e2. Use practice's next value<br>e3. Use population's next value	e1. Set to individual mode<br>e2. Set to practice mode<br>e3. Set to population mode	e1. Use individual's next value<br>e2. Use practice's next value<br>e3. Use population's next value	c(6). Set to 6 months if > 6 months
	f1. Use individual's next value<br>f2. Use practice's next value<br>f3. Use population's next value	f1. Use individual's previous value<br>f2. Use practice's previous value<br>f3. Use population's previous value	f1. Use individual's next value<br>f2. Use practice's next value<br>f3. Use population's next value	f1. Use individual's previous value<br>f2. Use practice's previous value<br>f3. Use population's previous value	c(12). Set to 12 months if >12 months
	g1. Use individual's previous value<br>g2. Use practice's previous value<br>g3. Use population's previous value		g1. Use individual's previous value<br>g2. Use practice's previous value<br>g3. Use population's previous value		c(24). Set to 24 months if >24months

Step:	B. Define prescription length		C. Handle concurrent & sequential prescriptions		
Decision node:	6<br>Generate stop dates	7<br>Clean missing stop dates**	8<br>Handle multiple prescriptions	9<br>Handle overlapping prescriptions	10<br>Handle gaps between prescriptions
	a. Start + numdays	a. Keep as missing	**a. Do nothing**	a. Ignore overlap	a. Do nothing: allow gap
	b. Start + dose_duration	b. Set to individual mean	b. Use mean ndd & duration	**b. Add overlap to end of 2nd prescription**	**b(15). Assume continuous use if gap < 15 days**
	c. Start + qty/ndd	c. Set to population mean	c. Use prescription with smallest ndd		b(30). Assume continuous use if gap < 30 days
	d(15). *Use mean if gap<15 days	**d. Use population mean if individual mean is missing**	d. Use prescription with largest ndd		b(60). Assume continuous use if gap < 60 days
	**d(30). *Use mean if gap<30 days**		e. Use shortest prescription		
	d(60). *Use mean if gap<60 days		f. Use longest prescription		
	d(90). *Use mean if gap<90 days		g. Sum durations		
	e. *Use mean regardless of gap				

qty = total quantity entered by GP for the prescribed product; ndd = derived numeric daily dose; numdays = number of treatment days; dose_duration = derived duration of prescription. All options that produce a missing value stay coded as missing unless otherwise stated.
*For options 6d-e: If only one is stop available, use it; if two are available and equal, use that date; if two available and unequal (but within X days), use mean; if three are available and unequal, use mean of closest two if within X days. **Records with missing stop dates after step 7 are dropped.

Variable list and details

Covariates included demographic and lifestyle characteristics, comorbidities in the Charlson comorbidity index[2,3] and the QRisk score,[4] and prescriptions for other medicines.

Code lists

Read code lists and drug lists are available on Zenodo (https://doi.org/10.5281/zenodo.4779024) and the ClinicalCodes repository (https://clinicalcodes.rss.mhs.man.ac.uk). New code lists were developed if a list was not available in the ClinicalCodes repository,[5] the CALIBER phenotyping resource,[6] or individual papers. To develop a new list, an initial list of terms was extracted based on key word searches and then screened by clinicians in the research team.

The depression code list was based on existing published lists[7,8] and the Quality and Outcomes Framework (QOF) business rules code release[9].

Demographic and lifestyle characteristics

Age in index year (based on year of birth), sex, ethnicity (using the England and Wales 2001 Census broad ethnicity categories – Asian or Asian British, Black or Black British, Mixed, Chinese or other ethnic group, White – most recent code prior to index date)[10], socio-economic status (SES, quintile of Townsend Score)[11], and practice region. Ethnicity was defined using Read-coded information in the primary care record, supplemented with available secondary care information if missing. Body mass index (BMI) was defined using recorded weight and height measurements. The most recent BMI, smoking status (never/ former/ current)[12], and alcohol use status (non-/ former/ occasional/ moderate/ heavy drinker)[13] on or prior to index date were defined.

Prescription medicine

Use of the following drugs were considered present if prescribed on or less than 6 months before index date: opioids, glucocorticoids, non-steroidal anti-inflammatory drugs (NSAIDs), other analgesics, statins, antipsychotics, anxiolytics, hypnotic agents.

Comorbidities

The following comorbidities were considered present if recorded in the primary care data on or before the index date: abdominal pain, inflammatory bowel disease, indigestion, liver disease, obesity, pancreatitis, peptic ulcer disease, renal disease, anaemia, atrial fibrillation, angina, cerebrovascular disease, congestive heart failure, diabetes mellitus, hypertension, myocardial infarction,[14] peripheral vascular disease, venous thromboembolism, alcohol misuse, anxiety,[15] contact with mental health services, eating disorder, insomnia,[16] intellectual disability, personality disorder, self-harm, substance misuse, appetite loss, living in a care home, hemiplegia, leg ulcer, palliative/end-of-life care, poor mobility, unexplained/unintentional weight loss, unplanned hospital admission, asthma, chronic obstructive pulmonary disease, dyspnoea,[17] sleep apnoea, acquired immunodeficiency syndrome (AIDS), cancer, recent cancer (past year), metastatic tumour, dementia, epilepsy,[18] fibromyalgia and generalised pain, Huntington's disease,[19] migraine, multiple sclerosis, neuropathic pain, Parkinson's disease, and rheumatological disease. An indicator of a hospital record for intentional self-harm was also defined.

Other variables

Depression severity was defined as having a coded record of severe depression or depression with psychosis, scoring 15 or above on the Patient Health Questionnaire-9 (PHQ-9) scale, or scoring 16 or above on the Hospital Anxiety and Depression (HAD) scale. An indicator of having a record of completing a depression scale was defined. Other variables were: most recent dose of the first antidepressant on/prior to index date, current dose of first antidepressant at index date (including 0), whether or not the first antidepressant was still being prescribed at index date, the time between starting the first and second antidepressants, the first SSRI prescribed, and the calendar year of index date.

Propensity score model
Variables associated with the exposure and outcome and variables associated with the outcome only (in univariate or age-sex adjusted models) were used to estimate propensity scores.[20]

Variables included in the propensity score model: age, sex, age-squared, body mass index, smoking status, alcohol intake, antipsychotics, anxiolytics, hypnotic agents, statins, substance misuse disorder, baseline self-harm (primary care records), peptic ulcer disease, pancreatitis, contact with mental health services, liver disease (mild), intellectual disability, insomnia, indigestion, hypertension, diabetes, cancer, anxiety, asthma, appetite loss, alcohol misuse.

Sensitivity analyses including all defined variables were also performed. Some variables were excluded as the models were not converging. Excluded variables were: AIDs, moderate liver disease, and Huntington's disease.

Additional results

Supplementary Table S1. Propensity score model
Multinomial logistic regression predicting study group based on baseline characteristics, with mirtazapine as the reference group. NOTE – this is an example showing one of the twenty imputed datasets. The results have not been combined at this stage.

	SSRI		Amitriptyline		Venlafaxine	
	RRR (95% CI)	p-value	RRR (95% CI)	p-value	RRR (95% CI)	p-value
Body mass index [a]	1.01 (1.01-1.02)	<0.001	1.03 (1.03-1.04)	<0.001	1.03 (1.02-1.04)	<0.001
Smoking status (vs. never smoker) [a]						
Former smoker	1.1 (1.01-1.2)	0.028	1.09 (0.98-1.22)	0.108	1.02 (0.88-1.18)	0.800
Current smoker	0.87 (0.8-0.95)	0.001	0.94 (0.84-1.05)	0.251	0.74 (0.64-0.86)	<0.001
Alcohol intake (vs. non-drinker) [a]						
Former drinker	0.92 (0.82-1.02)	0.106	0.96 (0.84-1.1)	0.555	0.88 (0.73-1.06)	0.189
Occasional drinker	0.98 (0.91-1.06)	0.604	0.99 (0.9-1.1)	0.846	0.97 (0.85-1.11)	0.697
Moderate drinker	0.98 (0.84-1.16)	0.853	0.88 (0.71-1.09)	0.237	1.03 (0.78-1.35)	0.839
Heavy drinker	0.95 (0.79-1.14)	0.573	0.83 (0.64-1.07)	0.145	0.87 (0.62-1.22)	0.433
Sex (female vs. male)	1.51 (1.41-1.61)	<0.001	2.1 (1.92-2.31)	<0.001	1.15 (1.02-1.3)	0.020
Age	1 (0.99-1.01)	0.908	1.09 (1.08-1.11)	<0.001	1.06 (1.04-1.08)	<0.001
Age squared	1 (1-1)	0.001	1 (1-1)	<0.001	1 (1-1)	<0.001
Antipsychotics	0.75 (0.64-0.89)	0.001	0.76 (0.61-0.94)	0.011	1.68 (1.33-2.13)	<0.001
Anxiolytics	0.72 (0.64-0.8)	<0.001	0.79 (0.68-0.91)	0.001	0.99 (0.83-1.19)	0.942
Hypnotic agents	0.56 (0.52-0.61)	<0.001	0.52 (0.46-0.58)	<0.001	0.76 (0.65-0.89)	<0.001
Statins	1.09 (0.97-1.24)	0.160	1.16 (1-1.35)	0.044	0.78 (0.61-1.01)	0.056
Substance misuse disorder	0.69 (0.57-0.84)	<0.001	0.78 (0.58-1.05)	0.101	0.65 (0.44-0.96)	0.029
Self-harm (primary care)	0.92 (0.77-1.1)	0.354	1.09 (0.85-1.4)	0.497	1.07 (0.79-1.44)	0.676
Peptic ulcer disease	1.03 (0.72-1.47)	0.862	1.13 (0.73-1.73)	0.589	0.89 (0.43-1.83)	0.745
Pancreatitis	2.23 (1.14-4.39)	0.020	2.78 (1.32-5.88)	0.007	1.4 (0.44-4.47)	0.568
Contact with mental health services	0.65 (0.6-0.7)	<0.001	0.56 (0.5-0.62)	<0.001	0.97 (0.85-1.1)	0.651
Liver disease (mild)	0.77 (0.38-1.54)	0.453	0.92 (0.39-2.13)	0.838	1.19 (0.38-3.74)	0.764
Intellectual disability	2.7 (1.28-5.73)	0.009	1.87 (0.71-4.94)	0.208	1.3 (0.39-4.36)	0.672
Insomnia	0.75 (0.68-0.83)	<0.001	1.25 (1.11-1.42)	<0.001	0.83 (0.69-1)	0.047
Indigestion	0.96 (0.89-1.03)	0.289	1.19 (1.08-1.3)	<0.001	0.94 (0.83-1.07)	0.364
Hypertension	0.95 (0.85-1.07)	0.383	0.95 (0.83-1.09)	0.445	0.74 (0.59-0.92)	0.007
Diabetes	0.9 (0.76-1.05)	0.182	1.07 (0.89-1.29)	0.497	1.08 (0.8-1.45)	0.627
Cancer	0.99 (0.85-1.14)	0.865	1.15 (0.96-1.37)	0.128	1.13 (0.86-1.48)	0.379
Anxiety	1.03 (0.95-1.11)	0.488	0.97 (0.88-1.07)	0.606	1.05 (0.93-1.19)	0.440
Asthma	0.92 (0.83-1.01)	0.092	1.21 (1.07-1.37)	0.003	0.98 (0.82-1.16)	0.8
Appetite loss	0.87 (0.67-1.14)	0.311	0.85 (0.6-1.21)	0.378	0.89 (0.53-1.5)	0.665
Alcohol misuse	0.84 (0.69-1.02)	0.083	0.79 (0.6-1.05)	0.107	0.72 (0.49-1.05)	0.091
Constant term	3.82 (2.93-4.98)	<0.001	0.03 (0.02-0.04)	<0.001	0.07 (0.04-0.12)	<0.001

This is an example from one of 20 multiple-imputed datasets as the results were combined at a later stage of the analysis. RRR relative risk ratio; CI confidence interval.

[a] Includes imputed data.

Supplementary Table S2. Full baseline characteristics

	All	Mirtazapine	SSRI	Amitriptyline	Venlafaxine	Statistic
**Count**	24,516	4,777	14,428	3,801	1,510	
**Age, median (IQR)**	41 (29-54)	44 (31-59)	39 (27-51)	48 (37-61)	41 (30-51)	KW chi2(3) = 890.9, p<0.001
**Sex, n(%)**						
Male	10,190 (41.6%)	2,456 (51.4%)	5,731 (39.7%)	1,303 (34.3%)	700 (46.4%)	
Female	14,326 (58.4%)	2,321 (48.6%)	8,697 (60.3%)	2,498 (65.7%)	810 (53.6%)	chi2(3) = 308.3, p<0.001
**Ethnicity, n(%)** [a]						
Asian	447 (2.5%)	105 (2.9%)	237 (2.3%)	83 (2.8%)	22 (2.1%)	
Black	259 (1.5%)	37 (1.0%)	150 (1.5%)	56 (1.9%)	16 (1.5%)	
Mixed	159 (0.9%)	35 (1.0%)	96 (0.9%)	18 (0.6%)	10 (1.0%)	
Other	214 (1.2%)	34 (1.0%)	136 (1.3%)	32 (1.1%)	12 (1.1%)	
White	16,728 (93.9%)	3,350 (94.1%)	9,614 (94.0%)	2,773 (93.6%)	991 (94.3%)	chi2(12) = 21.1, p=0.049
Missing ethnicity, n(%)	6,709 (27.4%)	1,216 (25.5%)	4,195 (29.1%)	839 (22.1%)	459 (30.4%)	chi2(3) = 90.5, p<0.001
**Townsend quintile, n(%)** [a]						
1 (least deprived)	4,830 (19.7%)	861 (18.0%)	2,853 (19.8%)	795 (20.9%)	321 (21.3%)	
2	4,962 (20.3%)	941 (19.7%)	2,898 (20.1%)	763 (20.1%)	360 (23.9%)	
3	5,290 (21.6%)	1,004 (21.0%)	3,118 (21.6%)	843 (22.2%)	325 (21.5%)	
4	5,305 (21.7%)	1,049 (22.0%)	3,186 (22.1%)	807 (21.2%)	263 (17.4%)	
5 (most deprived)	4,111 (16.8%)	918 (19.2%)	2,360 (16.4%)	593 (15.6%)	240 (15.9%)	chi2(12) = 60.1, p<0.001
**Missing Townsend score, n(%)**	30 (0.1%) [c]	<5	13 (0.1%)	<5	<5	chi2(3) = 3.4, p=0.331
**Region, n(%)**						
North East	500 (2.0%)	109 (2.3%)	276 (1.9%)	88 (2.3%)	27 (1.8%)	
North West	4,278 (17.4%)	1,184 (24.8%)	2,299 (15.9%)	570 (15.0%)	225 (14.9%)	
Yorkshire & The Humber	747 (3.0%)	118 (2.5%)	473 (3.3%)	127 (3.3%)	29 (1.9%)	
East Midlands	633 (2.6%)	90 (1.9%)	420 (2.9%)	105 (2.8%)	18 (1.2%)	
West Midlands	3,060 (12.5%)	496 (10.4%)	1,883 (13.1%)	435 (11.4%)	246 (16.3%)	
East of England	2,212 (9.0%)	334 (7.0%)	1,297 (9.0%)	378 (9.9%)	203 (13.4%)	
South West	3,396 (13.9%)	736 (15.4%)	1,846 (12.8%)	583 (15.3%)	231 (15.3%)	
South Central	3,162 (12.9%)	540 (11.3%)	1,871 (13.0%)	612 (16.1%)	139 (9.2%)	
London	2,534 (10.3%)	460 (9.6%)	1,561 (10.8%)	371 (9.8%)	142 (9.4%)	

Joseph RM, *et al. Evid Based Ment Health* 2022; 25:169–176. doi: 10.1136/ebmental-2021-300355

	All	Mirtazapine	SSRI	Amitriptyline	Venlafaxine	Statistic
South East Coast	3,994 (16.3%)	710 (14.9%)	2,502 (17.3%)	532 (14.0%)	250 (16.6%)	chi2(27) = 451.1, p<0.001
**BMI, median (IQR)** [a]	26.2 (22.8-30.8)	25.6 (22.4-29.8)	26.1 (22.7-30.5)	27.2 (23.5-32.2)	26.7 (23.2-31.2)	KW chi2(3) = 136.6, p<0.001
**Missing BMI, n(%)**	6,954 (28.4%)	1,409 (29.5%)	4,316 (29.9%)	794 (20.9%)	435 (28.8%)	chi2(3) = 124.7, p<0.001
**Smoking status, n(%)** [a]						
Never	9,635 (40.5%)	1,774 (38.3%)	5,725 (41.0%)	1,503 (40.1%)	633 (43.4%)	
Former	6,565 (27.6%)	1,243 (26.8%)	3,747 (26.8%)	1,173 (31.3%)	402 (27.6%)	
Current	7,600 (31.9%)	1,614 (34.9%)	4,496 (32.2%)	1,068 (28.5%)	422 (29.0%)	chi2(6) = 62.7, p<0.001
**Missing smoking status, n(%)**	716 (2.9%)	146 (3.1%)	460 (3.2%)	57 (1.5%)	53 (3.5%)	chi2(3) = 32.9, p<0.001
**Alcohol intake, n(%)** [a]						
Non-drinker	3,200 (33.4%)	615 (31.8%)	1,875 (34.3%)	534 (32.3%)	176 (34.1%)	
Former drinker	1,367 (14.3%)	316 (16.3%)	725 (13.3%)	263 (15.9%)	63 (12.2%)	
Occasional drinker	4,092 (42.7%)	790 (40.8%)	2,345 (42.9%)	733 (44.3%)	224 (43.4%)	
Moderate drinker	450 (4.7%)	98 (5.1%)	256 (4.7%)	71 (4.3%)	25 (4.8%)	
Heavy drinker	464 (4.8%)	118 (6.1%)	264 (4.8%)	54 (3.3%)	28 (5.4%)	chi2(12) = 36.8, p<0.001
**Missing alcohol intake, n(%)**	14,943 (61.0%)	2,840 (59.5%)	8,963 (62.1%)	2,146 (56.5%)	994 (65.8%)	chi2(3) = 60.1, p<0.001
**MENTAL HEALTH INDICATORS**						
Severe depression [b], n(%)	2,303 (9.4%)	472 (9.9%)	1,323 (9.2%)	327 (8.6%)	181 (12.0%)	chi2(3) = 16.9, p=0.001
Recorded depression scale, n(%)	15,076 (61.5%)	2,862 (59.9%)	8,879 (61.5%)	2,383 (62.7%)	952 (63.0%)	chi2(3) = 8.9, p=0.031
Alcohol misuse, n(%)	768 (3.1%)	220 (4.6%)	407 (2.8%)	99 (2.6%)	42 (2.8%)	chi2(3) = 42.9, p<0.001
Anxiety, n(%)	7,319 (29.9%)	1,458 (30.5%)	4,292 (29.7%)	1,090 (28.7%)	479 (31.7%)	chi2(3) = 6.1, p=0.106
Contact with mental health services, n(%)	5,895 (24.0%)	1,488 (31.1%)	3,264 (22.6%)	684 (18.0%)	459 (30.4%)	chi2(3) = 257.5, p<0.001
Eating disorder, n(%)	94 (0.4%)	17 (0.4%)	61 (0.4%)	9 (0.2%)	7 (0.5%)	chi2(3) = 3.1, p=0.380
Insomnia, n(%)	2,981 (12.2%)	769 (16.1%)	1,411 (9.8%)	621 (16.3%)	180 (11.9%)	chi2(3) = 208.1, p<0.001
Intellectual disability, n(%)	80 (0.3%) [c]	8 (0.2%)	58 (0.4%)	9 (0.2%)	<5	chi2(3) = 7.4, p=0.059
Personality disorder, n(%)	101 (0.4%)	24 (0.5%)	52 (0.4%)	15 (0.4%)	10 (0.7%)	chi2(3) = 4.2, p=0.239
Self-harm (primary care), n(%)	863 (3.5%)	185 (3.9%)	507 (3.5%)	110 (2.9%)	61 (4.0%)	chi2(3) = 7.3, p=0.062
Substance misuse disorder, n(%)	577 (2.4%)	177 (3.7%)	303 (2.1%)	65 (1.7%)	32 (2.1%)	chi2(3) = 49.2, p<0.001
**MEDICINES**						
Opioids, n(%)	4,169 (17.0%)	788 (16.5%)	1,772 (12.3%)	1,439 (37.9%)	170 (11.3%)	chi2(3) = 1,435.5, p<0.001

Joseph RM, *et al. Evid Based Ment Health* 2022; 25:169–176. doi: 10.1136/ebmental-2021-300355

	All	Mirtazapine	SSRI	Amitriptyline	Venlafaxine	Statistic
Glucocorticoids, n(%)	970 (4.0%)	219 (4.6%)	459 (3.2%)	248 (6.5%)	44 (2.9%)	chi2(3) = 98.1, p<0.001
NSAIDs, n(%)	3,598 (14.7%)	589 (12.3%)	1,741 (12.1%)	1,107 (29.1%)	161 (10.7%)	chi2(3) = 752.5, p<0.001
Other analgesics, n(%)	3,223 (13.1%)	660 (13.8%)	1,441 (10.0%)	1,000 (26.3%)	122 (8.1%)	chi2(3) = 738.6, p<0.001
Statins, n(%)	2,901 (11.8%)	675 (14.1%)	1,415 (9.8%)	689 (18.1%)	122 (8.1%)	chi2(3) = 245.6, p<0.001
Anxiolytics, n(%)	2,362 (9.6%)	635 (13.3%)	1,165 (8.1%)	379 (10.0%)	183 (12.1%)	chi2(3) = 125.0, p<0.001
Antipsychotics, n(%)	995 (4.1%)	274 (5.7%)	460 (3.2%)	149 (3.9%)	112 (7.4%)	chi2(3) = 106.5, p<0.001
Hypnotics, n(%)	3,894 (15.9%)	1,173 (24.6%)	1,893 (13.1%)	550 (14.5%)	278 (18.4%)	chi2(3) = 364.2, p<0.001
**COMORBIDITIES**						
Abdominal pain, n(%)	9,173 (37.4%)	1,683 (35.2%)	5,310 (36.8%)	1,669 (43.9%)	511 (33.8%)	chi2(3) = 88.7, p<0.001
Inflammatory bowel disease, n(%)	165 (0.7%)	31 (0.6%)	90 (0.6%)	39 (1.0%)	5 (0.3%)	chi2(3) = 10.3, p=0.016
Indigestion, n(%)	8,174 (33.3%)	1,614 (33.8%)	4,600 (31.9%)	1,487 (39.1%)	473 (31.3%)	chi2(3) = 74.1, p<0.001
Liver disease (mild), n(%)	55 (0.2%) [c]	14 (0.3%)	22 (0.2%)	10 (0.3%)	<5	chi2(3) = 4.7, p=0.198
Liver disease (moderate), n(%)	25 (0.1%) [c]	<5	8 (0.1%)	<5	<5	chi2(3) = 0.7, p=0.882
Obesity, n(%)	1,511 (6.2%)	250 (5.2%)	822 (5.7%)	340 (8.9%)	99 (6.6%)	chi2(3) = 63.8, p<0.001
Pancreatitis, n(%)	90 (0.4%) [c]	11 (0.2%)	49 (0.3%)	22 (0.6%)	<5	chi2(3) = 8.0, p=0.046
Peptic ulcer disease, n(%)	199 (0.8%)	50 (1.0%)	98 (0.7%)	42 (1.1%)	9 (0.6%)	chi2(3) = 11.4, p=0.010
Renal failure, n(%)	880 (3.6%)	234 (4.9%)	408 (2.8%)	197 (5.2%)	41 (2.7%)	chi2(3) = 79.1, p<0.001
Anaemia, n(%)	1,629 (6.6%)	346 (7.2%)	858 (5.9%)	334 (8.8%)	91 (6.0%)	chi2(3) = 43.1, p<0.001
Atrial fibrillation, n(%)	429 (1.7%)	135 (2.8%)	198 (1.4%)	83 (2.2%)	13 (0.9%)	chi2(3) = 55.2, p<0.001
Angina, n(%)	474 (1.9%)	127 (2.7%)	210 (1.5%)	122 (3.2%)	15 (1.0%)	chi2(3) = 70.3, p<0.001
Cerebrovascular disease, n(%)	435 (1.8%)	109 (2.3%)	219 (1.5%)	90 (2.4%)	17 (1.1%)	chi2(3) = 23.8, p<0.001
Congestive heart failure, n(%)	223 (0.9%)	58 (1.2%)	102 (0.7%)	56 (1.5%)	7 (0.5%)	chi2(3) = 28.2, p<0.001
Diabetes, n(%)	1,387 (5.7%)	303 (6.3%)	665 (4.6%)	345 (9.1%)	74 (4.9%)	chi2(3) = 118.8, p<0.001
Diabetes with complications, n(%)	320 (1.3%)	72 (1.5%)	133 (0.9%)	102 (2.7%)	13 (0.9%)	chi2(3) = 76.3, p<0.001
Hypertension, n(%)	3,020 (12.3%)	716 (15.0%)	1,488 (10.3%)	685 (18.0%)	131 (8.7%)	chi2(3) = 218.3, p<0.001
Myocardial infarction, n(%)	303 (1.2%)	80 (1.7%)	159 (1.1%)	57 (1.5%)	7 (0.5%)	chi2(3) = 19.2, p<0.001
Peripheral vascular disease, n(%)	244 (1.0%)	58 (1.2%)	117 (0.8%)	59 (1.6%)	10 (0.7%)	chi2(3) = 21.0, p<0.001
Venous thromboembolism, n(%)	226 (0.9%)	49 (1.0%)	119 (0.8%)	44 (1.2%)	14 (0.9%)	chi2(3) = 4.4, p=0.225
Appetite loss, n(%)	347 (1.4%)	92 (1.9%)	183 (1.3%)	54 (1.4%)	18 (1.2%)	chi2(3) = 11.7, p=0.008
Living in a care home, n(%)	60 (0.2%) [c]	21 (0.4%)	24 (0.2%)	8 (0.2%)	<5	chi2(3) = 16.0, p=0.001

Joseph RM, *et al. Evid Based Ment Health* 2022; 25:169–176. doi: 10.1136/ebmental-2021-300355

	All	Mirtazapine	SSRI	Amitriptyline	Venlafaxine	Statistic
Hemiplegia, n(%)	30 (0.1%) [c]	4 (0.1%)	14 (0.1%)	6 (0.2%)	<5	chi2(3) = 3.0, p=0.395
Leg ulcer, n(%)	172 (0.7%)	38 (0.8%)	79 (0.5%)	44 (1.2%)	11 (0.7%)	chi2(3) = 16.9, p=0.001
Palliative/end-of-life care, n(%)	100 (0.4%) [c]	29 (0.6%)	31 (0.2%)	33 (0.9%)	<5	chi2(3) = 39.6, p<0.001
Reduced mobility, n(%)	2,693 (11.0%)	692 (14.5%)	1,316 (9.1%)	549 (14.4%)	136 (9.0%)	chi2(3) = 163.7, p<0.001
Unexplained weight loss, n(%)	901 (3.7%)	226 (4.7%)	471 (3.3%)	148 (3.9%)	56 (3.7%)	chi2(3) = 22.4, p<0.001
Unexpected hospital admission, n(%)	1,446 (5.9%)	358 (7.5%)	724 (5.0%)	287 (7.6%)	77 (5.1%)	chi2(3) = 62.5, p<0.001
Asthma, n(%)	3,351 (13.7%)	630 (13.2%)	1,911 (13.2%)	604 (15.9%)	206 (13.6%)	chi2(3) = 19.0, p<0.001
Chronic obstructive pulmonary disease, n(%)	570 (2.3%)	142 (3.0%)	268 (1.9%)	137 (3.6%)	23 (1.5%)	chi2(3) = 54.4, p<0.001
Dyspnoea, n(%)	3,208 (13.1%)	676 (14.2%)	1,614 (11.2%)	742 (19.5%)	176 (11.7%)	chi2(3) = 191.7, p<0.001
Sleep apnoea, n(%)	197 (0.8%)	35 (0.7%)	95 (0.7%)	52 (1.4%)	15 (1.0%)	chi2(3) = 20.0, p<0.001
Acquired immune deficiency syndrome/ AIDS, n(%)	<20	<5	<5	<5	<5	chi2(3) = 1.4, p=0.706
Cancer, n(%)	1,362 (5.6%)	325 (6.8%)	667 (4.6%)	294 (7.7%)	76 (5.0%)	chi2(3) = 73.3, p<0.001
Recent cancer, n(%)	324 (1.3%)	76 (1.6%)	138 (1.0%)	90 (2.4%)	20 (1.3%)	chi2(3) = 49.3, p<0.001
Metastatic tumour, n(%)	35 (0.1%) [c]	12 (0.3%)	10 (0.1%)	8 (0.2%)	<5	chi2(3) = 11.5, p=0.009
Dementia, n(%)	223 (0.9%)	114 (2.4%)	73 (0.5%)	30 (0.8%)	6 (0.4%)	chi2(3) = 146.7, p<0.001
Epilepsy, n(%)	294 (1.2%)	74 (1.5%)	159 (1.1%)	44 (1.2%)	17 (1.1%)	chi2(3) = 6.2, p=0.102
Fibromyalgia, n(%)	562 (2.3%)	81 (1.7%)	256 (1.8%)	199 (5.2%)	26 (1.7%)	chi2(3) = 174.1, p<0.001
Huntington's disease, n(%)	<20	<5	5 (0.0%)	<5	<5	chi2(3) = 1.6, p=0.666
Migraine, n(%)	2,037 (8.3%)	366 (7.7%)	1,134 (7.9%)	434 (11.4%)	103 (6.8%)	chi2(3) = 59.1, p<0.001
Multiple sclerosis, n(%)	60 (0.2%) [c]	10 (0.2%)	16 (0.1%)	27 (0.7%)	<5	chi2(3) = 46.8, p<0.001
Neuropathic pain, n(%)	2,316 (9.4%)	427 (8.9%)	1,042 (7.2%)	743 (19.5%)	104 (6.9%)	chi2(3) = 549.8, p<0.001
Parkinson's disease, n(%)	80 (0.3%) [c]	28 (0.6%)	30 (0.2%)	13 (0.3%)	<5	chi2(3) = 17.8, p<0.001
Rheumatological disorders, n(%)	319 (1.3%)	75 (1.6%)	140 (1.0%)	88 (2.3%)	16 (1.1%)	chi2(3) = 46.1, p<0.001
**First SSRI [d], n(%)**						
Citalopram	12,613 (51.4%)	2,694 (56.4%)	7,030 (48.7%)	2,066 (54.4%)	823 (54.5%)	
Escitalopram	877 (3.6%)	135 (2.8%)	583 (4.0%)	102 (2.7%)	57 (3.8%)	
Fluoxetine	7,483 (30.5%)	1,162 (24.3%)	4,843 (33.6%)	1,055 (27.8%)	423 (28.0%)	
Paroxetine	285 (1.2%)	32 (0.7%)	205 (1.4%)	31 (0.8%)	17 (1.1%)	
Sertraline	3,258 (13.3%)	754 (15.8%)	1,767 (12.2%)	547 (14.4%)	190 (12.6%)	chi2(12) = 254.3, p<0.001

Joseph RM, *et al. Evid Based Ment Health* 2022; 25:169–176. doi: 10.1136/ebmental-2021-300355

	All	Mirtazapine	SSRI	Amitriptyline	Venlafaxine	Statistic
**Index year, median (IQR)**	2011 (2008-2013)	2011 (2009-2014)	2011 (2008-2013)	2011 (2009-2013)	2010 (2008-2013)	KW chi2(3) = 121.5, p<0.001
**Most recent antidepressant dose at index (DDD), median (IQR)**	1 (1-1)	1 (1-1.5)	1 (1-1)	1 (1-1)	1 (1-2)	KW chi2(3) = 253.1, p<0.001
**Current antidepressant dose at index (DDD), median (IQR)**	1 (0-1)	1 (0-1)	1 (0-1)	1 (0-1)	1 (0-1.5)	KW chi2(3) = 308.8, p<0.001
**Time (weeks) between starting first and second antidepressant, median (IQR)**	18.7 (6.1-75.9)	16.4 (6-65.6)	15.3 (5.1-61.9)	46.3 (12.1-138)	26.9 (10.1-87)	KW chi2(3) = 792.7, p<0.001
**First antidepressant still active at index, n(%)**	15,806 (64.5%)	3,294 (69.0%)	8,637 (59.9%)	2,847 (74.9%)	1,028 (68.1%)	chi2(3) = 364.8, p<0.001

SSRI selective serotonin reuptake inhibitor; IQR interquartile range; KW Kruskal-Wallis test; chi2() chi-square test (degrees of freedom); n number; BMI body mass index; DDD defined daily dose.

[a] Counts and percentages do not include missing values.

[b] Severe depression: Read code for severe depression or depression with psychosis, scoring 15 or above on the Patient Health Questionnaire-9 (PHQ-9) scale, or scoring 16 or above on the Hospital Anxiety and Depression (HAD) scale.

[c] Values have been rounded to mask small numbers.

[d] Small numbers (<5) initially prescribed fluvoxamine were combined with those initially prescribed citalopram.

Supplementary Table S3. Median follow-up per study group

	All	Mirtazapine	SSRI	Amitriptyline	Venlafaxine	Statistic
Follow-up time (months), median (interquartile range)	3.4 (1.9-12.1)	3.0 (1.9-9.7)	4.0 (1.9-14.1)	2.2 (1.9-5.2)	5.6 (2.0-21.3)	Kruskal-Wallis $Chi^2(3)=306$, $p<0.001$

SSRI selective serotonin reuptake inhibitor.

Joseph RM, *et al. Evid Based Ment Health* 2022; 25:169–176. doi: 10.1136/ebmental-2021-300355

Supplementary Table S4. Survival analyses comparing the risk of serious self-harm between study treatment groups while accounting for current antidepressant dose

	Age-sex adjusted, HR (95% CI)	Multivariable adjusted model, HR (95% CI)	PS weighted model, HR (95% CI)
Mirtazapine vs. SSRI	1.13 (0.68-1.86)	0.92 (0.55-1.52)	0.90 (0.51-1.59)
Mirtazapine vs. amitriptyline	2.45 (0.90-6.66)	1.97 (0.72-5.39)	1.70 (0.59-4.85)
Mirtazapine vs. venlafaxine	1.12 (0.51-2.46)	0.95 (0.43-2.08)	0.95 (0.40-2.27)
Mirtazapine current dose (DDD)	1.92 (1.21-3.05)	1.58 (0.99-2.52)	*1.88 (1.06-3.33)* [a]
SSRI current dose (DDD)	1.24 (1.07-1.43)	1.17 (1.02-1.35)	*1.23 (1.06-1.42)* [a]
Amitriptyline current dose (DDD)	0.32 (0.01-12.69)	0.28 (0.01-11.15)	*0.27 (0.01-7.94)* [a]
Venlafaxine current dose (DDD)	1.82 (1.23-2.69)	1.56 (1.06-2.29)	*1.73 (1.16-2.59)* [a]

HR hazard ratio, CI confidence interval, SSRI selective serotonin reuptake inhibitor, PS propensity score; DDD defined daily dose. Analyses performed using Cox regression. The multivariable adjusted model included all variables used to estimate the propensity scores. The PS weighted model applied inverse probability of treatment weights.

[a] These estimates will not be well adjusted for baseline characteristics: the propensity score only balances the probability of being prescribed each antidepressant.

Joseph RM, *et al. Evid Based Ment Health* 2022; 25:169–176. doi: 10.1136/ebmental-2021-300355

Supplementary Table S5. Sensitivity analyses – rates after altering study inclusion criteria

	Crude mortality rate (95% CI)	Age-sex Standardised mortality rate (95% CI)	Rate difference (95% CI)
**Excluding people with self-harm recorded at baseline**			
All	7.4 (6.4-8.5)	7.4 (6.4-8.5)	..
Mirtazapine	9.1 (6.6-12.4)	9.8 (6.7-13.7)	reference
SSRI	7.2 (6.0-8.6)	6.9 (5.7-8.3)	-2.9 (-4.5..1.3)
Amitriptyline	2.7 (1.3-5.4)	4.0 (1.6-7.8)	-5.9 (-7.3..4.4)
Venlafaxine	11.7 (7.9-17.4)	11.2 (7.1-17.5)	1.4 (-0.4-3.2)
**Include people with schizophrenia/ bipolar disorder recorded at baseline**			
All	8.9 (7.8-10.1)	8.9 (7.8-10.1)	..
Mirtazapine	12.8 (9.8-16.6)	14.1 (10.4-18.6)	reference
SSRI	8.5 (7.2-10.0)	8.1 (6.8-9.5)	-6.0 (-7.8..4.2)
Amitriptyline	2.6 (1.3-5.2)	3.8 (1.6-7.6)	-10.2 (-11.9..8.6)
Venlafaxine	12.5 (8.7-18.2)	12.0 (7.8-18.2)	-2.1 (-4.1..0.2)

CI confidence interval; SSRI selective serotonin reuptake inhibitor. Attributable risks shown are calculated as mirtazapine minus the comparator.

Supplementary Table S6. Sensitivity analysis – multivariable competing risks regression

	Subdistribution hazard ratio (95% CI)
Mirtazapine / SSRI	1.10 (0.79-1.53)
Mirtazapine / amitriptyline	3.21 (1.50-6.87)
Mirtazapine / venlafaxine	0.81 (0.50-1.31)
Body mass index	0.99 (0.96-1.01)
Smoking status (vs. never smoker)	
Former smoker	1.33 (0.93-1.91)
Current smoker	1.37 (0.99-1.91)
Alcohol intake (vs. non-drinker)	
Former drinker	0.92 (0.49-1.72)
Occasional drinker	1.19 (0.69-2.06)
Moderate drinker	1.08 (0.40-2.87)
Heavy drinker	1.76 (0.72-4.29)
Sex (female vs. male)	0.76 (0.59-0.99)
Age	0.97 (0.93-1.01)
Age squared	1.00 (1.00-1.00)
Antipsychotics	2.12 (1.33-3.36)
Anxiolytics	1.61 (1.12-2.32)
Hypnotic agents	2.05 (1.50-2.79)
Statins	1.17 (0.62-2.20)
Substance misuse disorder	1.14 (0.64-2.04)
Self-harm (primary care)	3.79 (2.60-5.54)
Peptic ulcer disease	0.88 (0.30-2.56)
Pancreatitis	3.78 (1.16-12.33)
Contact with mental health services	1.20 (0.90-1.61)
Liver disease (mild)	3.01 (0.81-11.21)
Intellectual disability	2.69 (0.67-10.78)
Insomnia	1.19 (0.82-1.72)
Indigestion	1.28 (0.98-1.68)
Hypertension	0.70 (0.37-1.31)
Diabetes	0.69 (0.27-1.78)
Cancer	0.64 (0.28-1.48)
Anxiety	1.30 (0.99-1.70)
Asthma	1.05 (0.73-1.49)
Appetite loss	2.61 (1.31-5.21)
Alcohol misuse	1.86 (0.97-3.58)

CI confidence interval; SSRI selective serotonin reuptake inhibitor.

Supplementary Table S7. Sensitivity analyses – varying the survival analysis models (all analyses used Cox regression)

				Hazard Ratio (95% confidence interval)		
Analysis / Model	Number of people	Number of deaths	Total person-years	Mirtazapine vs. SSRI	Mirtazapine vs. Amitriptyline	Mirtazapine vs. Venlafaxine
**Repeat main analysis using Cox regression instead of Fine-Gray regression**						
Unadjusted	24,516	235	26,679	1.38 (1.01-1.87)	5.10 (2.43-10.70)	0.86 (0.54-1.35)
Age-sex adjusted	24,516	235	26,679	1.52 (1.11-2.07)	4.34 (2.06-9.13)	0.94 (0.59-1.49)
Fully adjusted	24,516	235	26,679	1.10 (0.80-1.52)	3.21 (1.52-6.80)	0.82 (0.51-1.31)
Weighted	24,516	235	26,679	1.19 (0.85-1.67)	3.05 (1.37-6.81)	0.86 (0.52-1.42)
**Including all baseline covariates [a]**						
Fully adjusted	24,516	235	26,679	1.09 (0.78-1.51)	2.97 (1.39-6.36)	0.76 (0.47-1.23)
Weighted	24,516	235	26,679	1.16 (0.81-1.65)	3.11 (1.32-7.29)	0.77 (0.44-1.35)
**Excluding people with baseline primary care record of self-harm**						
Age-sex adjusted	23,653	191	25,911	1.22 (0.85-1.76)	3.02 (1.41-6.49)	0.69 (0.41-1.14)
Fully adjusted	23,653	191	25,911	0.90 (0.62-1.31)	2.26 (1.04-4.88)	0.60 (0.36-1.01)
**Restrict by age group: people aged 18-64 years at baseline**						
Age-sex adjusted	21,325	222	22,986	1.47 (1.06-2.04)	3.77 (1.78-7.99)	0.92 (0.57-1.48)
Fully adjusted	21,325	222	22,986	1.08 (0.77-1.51)	2.84 (1.33-6.06)	0.78 (0.48-1.27)
**Restrict by age group: people aged 65-99 years at baseline**						
Age-sex adjusted	3,191	13	3,693	2.29 (0.72-7.27)	..	1.16 (0.14-9.48)
Fully adjusted	3,191	13	3,693	1.99 (0.56-7.09)	..	1.20 (0.12-11.99)
**New follow-up rule: do not censor when a third antidepressant is prescribed**						
Age-sex adjusted	24,594	311	35,826	1.70 (1.30-2.22)	2.64 (1.58-4.40)	1.17 (0.77-1.78)
Fully adjusted	24,594	311	35,826	1.26 (0.96-1.65)	1.99 (1.18-3.35)	1.00 (0.65-1.52)
**New follow-up rule: exposure carry-over window of 0 days from antidepressant stop date**						
Age-sex adjusted	24,516	177	21,178	1.43 (1.00-2.06)	4.79 (1.89-12.17)	1.08 (0.61-1.90)
Fully adjusted	24,516	177	21,178	1.07 (0.74-1.55)	3.58 (1.40-9.17)	0.97 (0.55-1.74)
**New follow-up rule: exposure carry-over window of 6 months from antidepressant stop date**						
Age-sex adjusted	24,516	282	35,601	1.51 (1.13-2.01)	3.58 (1.97-6.51)	0.85 (0.56-1.29)
Fully adjusted	24,516	282	35,601	1.13 (0.84-1.51)	2.73 (1.49-4.98)	0.74 (0.48-1.13)
**New follow-up rule: ignore antidepressant end date, follow-up to study end, leaving cohort, or starting a third antidepressant**						
Age-sex adjusted	24,516	361	62,350	1.38 (1.07-1.78)	2.71 (1.71-4.28)	0.84 (0.57-1.25)
Fully adjusted	24,516	361	62,350	1.04 (0.80-1.36)	2.08 (1.31-3.31)	0.76 (0.51-1.13)

Joseph RM, *et al. Evid Based Ment Health* 2022; 25:169–176. doi: 10.1136/ebmental-2021-300355

Analysis / Model	Number of people	Number of deaths	Total person-years	Hazard Ratio (95% confidence interval)		
				Mirtazapine vs. SSRI	Mirtazapine vs. Amitriptyline	Mirtazapine vs. Venlafaxine
**New follow-up rule: censor follow-up after 5 years**						
Age-sex adjusted	24,516	231	16,952	1.53 (1.12-2.09)	4.31 (2.05-9.07)	1.01 (0.63-1.61)
Fully adjusted	24,516	231	16,952	1.12 (0.81-1.54)	3.20 (1.51-6.78)	0.87 (0.54-1.40)
**New follow-up rule: censor follow-up after 1 year**						
Age-sex adjusted	24,516	191	5,227	1.25 (0.88-1.78)	4.38 (1.86-10.33)	0.97 (0.56-1.69)
Fully adjusted	24,516	191	5,227	0.91 (0.63-1.31)	3.31 (1.39-7.87)	0.83 (0.47-1.45)
**Specific SSRIs: include only those who were initially prescribed citalopram**						
Age-sex adjusted	12,613	118	13,703	1.58 (1.04-2.40)	6.45 (1.97-21.10)	1.36 (0.69-2.69)
Fully adjusted	12,613	118	13,703	1.11 (0.72-1.71)	4.68 (1.42-15.48)	1.19 (0.59-2.39)
**Specific SSRIs: restrict the SSRI group to those prescribed citalopram as their 2nd antidepressant; exclude all people initially prescribed citalopram**						
Age-sex adjusted	9,293	84	10,070	1.68 (1.00-2.83)	2.99 (1.13-7.93)	0.67 (0.35-1.27)
Fully adjusted	9,293	84	10,070	1.37 (0.79-2.37)	2.25 (0.83-6.05)	0.54 (0.28-1.06)
**Broader outcome, including record for self-harm in the primary care data (dropping people with any self-harm record at index date)**						
Age-sex adjusted	23,653	330	25,731	1.21 (0.91-1.60)	1.84 (0.99-3.42)	0.75 (0.49-1.14)
Fully adjusted	23,653	330	25,731	1.47 (1.12-1.93)	2.56 (1.50-4.35)	0.83 (0.56-1.21)
Weighted	23,653	330	25,731	1.12 (0.84-1.48)	1.96 (1.15-3.35)	0.71 (0.48-1.05)

‘Fully adjusted’ models used multivariable regression, ‘weighted’ models used inverse probability of treatment weighting. SSRI selective serotonin reuptake inhibitors.

[a] Some variables were excluded as the models did not converge. Excluded variables were: AIDs, moderate liver disease, and Huntington’s disease.

Supplementary Table S8. Summary of antidepressant doses during follow-up (including times of non-use)

	Study treatment group			
Median (IQR) of median dose	Mirtazapine	SSRI	Amitriptyline	Venlafaxine
Mirtazapine	0.5 (0.0-1.0)	..	..	..
SSRI	0.0 (0.0-0.5)	1.0 (0.5-1.0)	1.0 (0.0-1.0)	0.0 (0.0-0.0)
Amitriptyline	..	..	0.1 (0.0-0.2)	..
Venlafaxine	..	..	..	0.8 (0.4-1.5)
All antidepressants	1.0 (0.5-1.5)	1.0 (0.5-1.0)	1.0 (0.2-1.2)	0.8 (0.8-1.5)

Median dose over follow-up was calculated for each person, including times of non-use (dose=0). The summary data are the median (interquartile range) of these values according to study treatment group. Doses are converted to defined daily dose (DDD). SSRI selective serotonin-reuptake inhibitor.

Supplementary Table S9. Summary of antidepressant doses during follow-up (excluding times of non-use)

	Study treatment group			
Median (IQR) of median dose	Mirtazapine	SSRI	Amitriptyline	Venlafaxine
Mirtazapine	0.5 (0.5-1.0)	..	..	..
SSRI	1.0 (0.0-1.0)	1.0 (1.0-2.0)	1.0 (0.5-1.0)	1.0 (0.0-2.0)
Amitriptyline	..	..	0.2 (0.1-0.3)	..
Venlafaxine	..	..	..	0.8 (0.8-1.5)
All antidepressants	1.0 (0.5-1.5)	1.0 (1.0-2.0)	1.0 (0.3-1.3)	1.3 (0.8-1.5)

Median dose over follow-up was calculated for each person, excluding times of non-use (dose=0). The summary data are the median (interquartile range) of these values according to study treatment group. Doses are converted to defined daily dose (DDD). SSRI selective serotonin-reuptake inhibitor.

Supplementary Table S10. Comparison of characteristics between those meeting the different inclusion criteria

	1) SSRI users with linked data	2) 1 & first antidepressant was an SSRI	3) 2 & people ever had a second antidepressant	4) 3 & the second antidepressant was prescribed within 90 days of a course of the first	5) 4 & people had a depression record in the required window	6) 5 & no exclusion criteria [c] (study cohort)
**Count**	358,911	271,868	83,271	44,781	25,820	24,516
**Age at first recorded antidepressant prescription, median (IQR)**	43 (31-58)	42 (29-57)	41 (28-55)	41 (28-55)	40 (27-52)	40 (28-53)
**Sex, n(%)**						
Male	137,009 (38.2%)	108,160 (39.8%)	31,449 (37.8%)	18,071 (40.4%)	10,723 (41.5%)	10,190 (41.6%)
Female	221,902 (61.8%)	163,708 (60.2%)	51,822 (62.2%)	26,710 (59.6%)	15,097 (58.5%)	14,326 (58.4%)
**Townsend quintile, n(%)** [a]						
1 (least deprived)	75,142 (21.0%)	57,439 (21.1%)	16,729 (20.1%)	8,917 (19.9%)	5,018 (19.5%)	4,830 (19.7%)
2	75,452 (21.0%)	57,389 (21.1%)	16,704 (20.1%)	9,057 (20.2%)	5,155 (20.0%)	4,962 (20.3%)
3	76,442 (21.3%)	58,008 (21.4%)	17,645 (21.2%)	9,543 (21.3%)	5,555 (21.5%)	5,290 (21.6%)
4	77,375 (21.6%)	58,368 (21.5%)	18,525 (22.3%)	9,839 (22.0%)	5,642 (21.9%)	5,305 (21.7%)
5 (most deprived)	54,182 (15.1%)	40,409 (14.9%)	13,603 (16.3%)	7,391 (16.5%)	4,428 (17.2%)	4,111 (16.8%)
**Missing Townsend score, n(%)**	318 (0.1%)	255 (0.1%)	65 (0.1%)	34 (0.1%)	22 (0.1%)	18 (0.1%)
**Region, n(%)**						
North East	8,523 (2.4%)	5,892 (2.2%)	1,885 (2.3%)	1,071 (2.4%)	535 (2.1%)	500 (2.0%)
North West	60,713 (16.9%)	44,483 (16.4%)	14,617 (17.6%)	7,824 (17.5%)	4,568 (17.7%)	4,278 (17.4%)
Yorkshire & The Humber	13,204 (3.7%)	9,253 (3.4%)	2,734 (3.3%)	1,485 (3.3%)	784 (3.0%)	747 (3.0%)
East Midlands	9,164 (2.6%)	6,940 (2.6%)	1,550 (1.9%)	918 (2.0%)	656 (2.5%)	633 (2.6%)
West Midlands	43,157 (12.0%)	33,512 (12.3%)	10,254 (12.3%)	5,442 (12.2%)	3,220 (12.5%)	3,060 (12.5%)
East of England	34,760 (9.7%)	25,697 (9.5%)	7,253 (8.7%)	3,992 (8.9%)	2,300 (8.9%)	2,212 (9.0%)
South West	48,251 (13.4%)	35,791 (13.2%)	11,348 (13.6%)	6,074 (13.6%)	3,599 (13.9%)	3,396 (13.9%)
South Central	48,591 (13.5%)	36,129 (13.3%)	11,142 (13.4%)	6,023 (13.4%)	3,315 (12.8%)	3,162 (12.9%)
London	36,399 (10.1%)	29,690 (10.9%)	8,655 (10.4%)	4,699 (10.5%)	2,660 (10.3%)	2,534 (10.3%)
South East Coast	56,149 (15.6%)	44,481 (16.4%)	13,833 (16.6%)	7,253 (16.2%)	4,183 (16.2%)	3,994 (16.3%)
**Age at index date, median (IQR)**			43 (30-57)	42 (29-56)	41 (29-54)	41 (29-54)
**Smoking status, n(%)** [a]						
Never			32,377 (39.8%)	17,697 (40.7%)	10,016 (40.0%)	9,635 (40.5%)

Joseph RM, *et al. Evid Based Ment Health* 2022; 25:169–176. doi: 10.1136/ebmental-2021-300355

	1) SSRI users with linked data	2) 1 & first antidepressant was an SSRI	3) 2 & people ever had a second antidepressant	4) 3 & the second antidepressant was prescribed within 90 days of a course of the first	5) 4 & people had a depression record in the required window	6) 5 & no exclusion criteria [c] (study cohort)
Former			23,555 (29.0%)	12,361 (28.4%)	6,809 (27.2%)	6,565 (27.6%)
Current			25,341 (31.2%)	13,392 (30.8%)	8,238 (32.9%)	7,600 (31.9%)
**Missing smoking status, n(%)**			1,998 (2.4%)	1,331 (3.0%)	757 (2.9%)	716 (2.9%)
**Ethnicity, n(%)** [a]						
Asian			1,627 (2.5%)	783 (2.3%)	467 (2.5%)	447 (2.5%)
Black			862 (1.3%)	445 (1.3%)	280 (1.5%)	259 (1.5%)
Mixed			508 (0.8%)	271 (0.8%)	168 (0.9%)	159 (0.9%)
Other			693 (1.1%)	411 (1.2%)	219 (1.2%)	214 (1.2%)
White			60,573 (94.3%)	31,585 (94.3%)	17,845 (94.0%)	16,728 (93.9%)
**Missing ethnicity, n(%)**			19,008 (22.8%)	11,286 (25.2%)	6,841 (26.5%)	6,709 (27.4%)
**BMI, median (IQR)** [a]			26.1 (22.7-30.4)	26.0 (22.7-30.3)	26.2 (22.8-30.7)	26.2 (22.8-30.8)
Missing BMI, n(%)			22,175 (26.6%)	12,721 (28.4%)	7,396 (28.6%)	6,954 (28.4%)
**Alcohol intake, n(%)** [a]						
Non-drinker			11,321 (33.5%)	6,073 (34.5%)	3,352 (33.3%)	3,200 (33.4%)
Former drinker			4,959 (14.7%)	2,563 (14.6%)	1,468 (14.6%)	1,367 (14.3%)
Occasional drinker			14,292 (42.2%)	7,307 (41.5%)	4,254 (42.2%)	4,092 (42.7%)
Moderate drinker			1,532 (4.5%)	809 (4.6%)	470 (4.7%)	450 (4.7%)
Heavy drinker			1,725 (5.1%)	842 (4.8%)	531 (5.3%)	464 (4.8%)
**Missing alcohol intake, n(%)**			49,442 (59.4%)	27,187 (60.7%)	15,745 (61.0%)	14,943 (61.0%)
**Index year, median (IQR)**			2011 (2009-2014)	2011 (2008-2013)	2011 (2008-2013)	2011 (2008-2013)
**Most recent antidepressant dose at index (DDD), median (IQR)**			1 (1-1)	1 (1-1)	1 (1-1)	1 (1-1)
**Current antidepressant dose at index (DDD), median (IQR)**			0 (0-1)	1 (0-1)	1 (0-1)	1 (0-1)
**Time (weeks) between starting first and second antidepressant, median (IQR)**			54.7 (12.3-151.3)	15.1 (5.1-60)	19 (6.3-76.6)	18.7 (6.1-75.9)
**First antidepressant still active at index, n(%)**			30,218 (36.3%)	28,549 (63.8%)	16,584 (64.2%)	15,806 (64.5%)
**MENTAL HEALTH INDICATORS**						
Severe depression [b], n(%)			5,257 (6.3%)	2,684 (6.0%)	2,447 (9.5%)	2,303 (9.4%)

	1) SSRI users with linked data	2) 1 & first antidepressant was an SSRI	3) 2 & people ever had a second antidepressant	4) 3 & the second antidepressant was prescribed within 90 days of a course of the first	5) 4 & people had a depression record in the required window	6) 5 & no exclusion criteria [c] (study cohort)
Recorded depression scale, n(%)			38,965 (46.8%)	20,295 (45.3%)	15,831 (61.3%)	15,076 (61.5%)
Alcohol misuse, n(%)			2,933 (3.5%)	1,481 (3.3%)	910 (3.5%)	768 (3.1%)
Anxiety, n(%)			23,317 (28.0%)	12,407 (27.7%)	7,744 (30.0%)	7,319 (29.9%)
Contact with mental health services, n(%)			19,584 (23.5%)	10,939 (24.4%)	6,612 (25.6%)	5,895 (24.0%)
Eating disorder, n(%)			445 (0.5%)	229 (0.5%)	120 (0.5%)	94 (0.4%)
Insomnia, n(%)			10,207 (12.3%)	5,184 (11.6%)	3,171 (12.3%)	2,981 (12.2%)
Intellectual disability, n(%)			361 (0.4%)	228 (0.5%)	90 (0.3%)	79 (0.3%)
Personality disorder, n(%)			457 (0.5%)	254 (0.6%)	146 (0.6%)	101 (0.4%)
Self-harm (primary care), n(%)			4,653 (5.6%)	2,543 (5.7%)	1,697 (6.6%)	863 (3.5%)
Substance misuse disorder, n(%)			2,272 (2.7%)	1,163 (2.6%)	667 (2.6%)	577 (2.4%)

SSRI selective serotonin reuptake inhibitor; IQR interquartile range; n number; BMI body mass index; DDD defined daily dose. For columns 1) and 2), only a subset of people had an index date, hence certain results are omitted for these columns. Characteristics defined with respect to index date if not otherwise specified.

[a] Counts and percentages do not include missing values.

[b] Severe depression: Read code for severe depression or depression with psychosis, scoring 15 or above on the Patient Health Questionnaire-9 (PHQ-9) scale, or scoring 16 or above on the Hospital Anxiety and Depression (HAD) scale.

[c] Exclusion criteria: aged 100 years or more, record for schizophrenia, record for bipolar disorder, or hospital record for self-harm at index.

Supplementary Table S11. Number of active antidepressant prescriptions at follow-up=3months, by study treatment group

Number of antidepressants	Mirtazapine	SSRI	Amitriptyline	Venlafaxine	Total
0	504 (19.3%)	1665 (18.2%)	271 (17.1%)	131 (12.7%)	2571 (17.8%)
1	1813 (69.4%)	7215 (79.0%)	688 (43.5%)	856 (82.7%)	10,572 (73.6%)
2	297 (11.4%)	251 (2.8%)	628 (39.6%)	48 (4.6%)	1224 (8.5%)
Total count	2614	9131	1587	1035	14,367

There were 14,367 people still in the cohort after 84 days of follow-up. This represents 58.6% of the total cohort, 54.7% of the mirtazapine group, 63.3% of the SSRI group, 41.7% of the amitriptyline group, and 68.5% of the venlafaxine group. People still prescribed their initial antidepressant alongside the treatment of interest at follow-up=84 days will have a total antidepressant count of 2. Those who have stopped both treatments have an antidepressant count of 0. Values shown are number (column %). SSRI selective serotonin-reuptake inhibitor.

Joseph RM, *et al. Evid Based Ment Health* 2022; 25:169–176. doi: 10.1136/ebmental-2021-300355

References

1. Pye SR, Sheppard T, Joseph RM, et al. Assumptions made when preparing drug exposure data for analysis have an impact on results: An unreported step in pharmacoepidemiology studies. *Pharmacoepidemiol Drug Saf* 2018; **27**(7): 781-8.
2. Charlson ME, Pompei P, Ales KL, MacKenzie CR. A new method of classifying prognostic comorbidity in longitudinal studies: development and validation. *J Chronic Dis* 1987; **40**(5): 373-83.
3. Khan NF, Perera R, Harper S, Rose PW. Adaptation and validation of the Charlson Index for Read/OXMIS coded databases. *BMC Fam Pract* 2010; **11**(1): 1.
4. Hippisley-Cox J, Coupland C. Development and validation of QMortality risk prediction algorithm to estimate short term risk of death and assess frailty: cohort study. *BMJ* 2017; **358**: j4208.
5. Springate DA, Kontopantelis E, Ashcroft DM, et al. ClinicalCodes: an online clinical codes repository to improve the validity and reproducibility of research using electronic medical records. *PLoS One* 2014; **9**(6): e99825.
6. Denaxas S, Gonzalez-Izquierdo A, Direk K, et al. UK phenomics platform for developing and validating electronic health record phenotypes: CALIBER. *J Am Med Inform Assoc* 2019; **26**(12): 1545-59.
7. John A, McGregor J, Fone D, et al. Case-finding for common mental disorders of anxiety and depression in primary care: an external validation of routinely collected data. *BMC Med Inform Decis Mak* 2016; **16**(1): 35.
8. Carreira H, Williams R, Strongman H, Bhaskaran K. Identification of mental health and quality of life outcomes in primary care databases in the UK: a systematic review. *BMJ Open* 2019; **9**(7): e029227.
9. NHS Digital. Quality and Outcomes Framework (QOF) business rules v 38 2017-2018 October code release. 2018. https://digital.nhs.uk/data-and-information/data-collections-and-data-sets/data-collections/quality-and-outcomes-framework-qof/quality-and-outcome-framework-qof-business-rules/quality-and-outcomes-framework-qof-business-rules-v-38-2017-2018-october-code-release09 Dec 2021).
10. Wright AK, Kontopantelis E, Emsley R, et al. Life Expectancy and Cause-Specific Mortality in Type 2 Diabetes: A Population-Based Cohort Study Quantifying Relationships in Ethnic Subgroups. *Diabetes Care* 2017; **40**(3): 338-45.
11. Townsend P. Deprivation. *Journal of Social Policy* 1987; **16**: 125-46.
12. Joseph RM, Movahedi M. SmokingDefinition (STATA): An algorithm to define smoking status in patients of the UK Clinical Practice Research Datalink (CPRD) (Version v1.3). *Zenodo* 2018; (http://doi.org/10.5281/zenodo.1405937).
13. Bell S, Daskalopoulou M, Rapsomaniki E, et al. Association between clinically recorded alcohol consumption and initial presentation of 12 cardiovascular diseases: population based cohort study using linked health records. *BMJ* 2017; **356**: j909.
14. Morgan C, Webb RT, Carr MJ, et al. Self-harm in a primary care cohort of older people: incidence, clinical management, and risk of suicide and other causes of death. *Lancet Psychiatry* 2018; **5**(11): 905-12.
15. John A, McGregor J, Fone D, et al. Case-finding for common mental disorders of anxiety and depression in primary care: an external validation of routinely collected data. *BMC Med Inform Decis Mak* 2016; **16**: 35.
16. Hoile R, Tabet N, Smith H, Bremner S, Cassell J, Ford E. Are symptoms of insomnia in primary care associated with subsequent onset of dementia? A matched retrospective case-control study. *Aging Ment Health* 2020; **24**(9): 1466-71.
17. Watson J, Nicholson BD, Hamilton W, Price S. Identifying clinical features in primary care electronic health record studies: methods for codelist development. *BMJ Open* 2017; **7**(11): e019637.
18. Gorton HC, Webb RT, Carr MJ, DelPozo-Banos M, John A, Ashcroft DM. Risk of Unnatural Mortality in People With Epilepsy. *JAMA Neurol* 2018; **75**(8): 929-38.
19. Wexler NS, Collett L, Wexler AR, et al. Incidence of adult Huntington's disease in the UK: a UK-based primary care study and a systematic review. *BMJ Open* 2016; **6**(2): e009070.
20. Brookhart MA, Schneeweiss S, Rothman KJ, Glynn RJ, Avorn J, Sturmer T. Variable selection for propensity score models. *Am J Epidemiol* 2006; **163**(12): 1149-56.
